# Supplementary material for: Astrocytic Ca2+ signaling is reduced during sleep and is involved in the regulation of slow wave sleep
Source: Nat Commun. 2020 Jul 6;11:3240. doi: 10.1038/s41467-020-17062-2 (PMC7338360; doi:10.1038/s41467-020-17062-2)
Supplement: Supplementary file 1 — Reporting Summary [file 41467_2020_17062_MOESM1_ESM.pdf]

Corresponding Author Name: Rune Enger

Manuscript Number: NCOMMS-19-539500A-Z

Reporting Checklist For Nature Communications Life Sciences Articles

This checklist is used to ensure good reporting standards and to improve the reproducibility of published results. For more information, please read Reporting Life Sciences Research.

Figure legends

☑ Check here to confirm that the following information is available in all relevant figure legends (or Methods section if too long):

- the **exact sample size (*n*)** for each experimental group/condition, given as a number, not a range;
- a **description of the sample collection** allowing the reader to understand whether the samples represent **technical or biological replicates** (including how many animals, litters, culture, etc.);
- a **statement of how many times the experiment shown was replicated in the laboratory**;
- **definitions of statistical methods and measures**: (For small sample sizes (n<5) descriptive statistics are not appropriate, instead plot individual data points)
  - very common tests, such as *t*-test, simple χ^2 tests, Wilcoxon and Mann-Whitney tests, can be unambiguously identified by name only, but more complex techniques should be described in the methods section;
  - are tests one-sided or two-sided?
  - are there adjustments for multiple comparisons?
  - **statistical test results**, e.g., ***P* values**;
  - definition of '**center values**' as **median** or **mean**;
  - definition of **error bars** as **s.d.** or **s.e.m.** or **c.i.**

This checklist will not be published. Please ensure that the answers to the following questions are reported in the manuscript itself. We encourage you to include a specific subsection in the Methods section for statistics, reagents and animal models. Below, provide the page number or section and paragraph number (e.g. "Page 5" or "Methods, 'reagents' subsection, paragraph 2").

Statistics and general methods	Reported in section/paragraph or page #:
How was the sample size chosen to ensure adequate power to detect a pre-specified effect size? (Give section/paragraph or page #)	Methods section"Animals", page 18. Sample sizes chosen based on typical sample sizes in the litterature and previous studies.
For animal studies, include a statement about sample size estimate even if no statistical methods were used.	Methods section "Animals", page 18. No power calculations were performed to calculate sample sizes.
Describe inclusion/exclusion criteria if samples or animals were excluded from the analysis. Were the criteria pre-established? (Give section/paragraph or page #)	Methods section "Surgical procedures and intrinsic imaging", page 16. Methods section "Behavioral training", page 20. Mice with surgical complications or that did not accomodate to head-fixation were excluded from the study.
If a method of randomization was used to determine how samples/animals were allocated to experimental groups and processed, describe it. (Give section/paragraph or page #)	Methods section "Animals", page 18. No randomization of mice into different experimental groups were performed. Our groups consisted of different genotypes, and not the same genotype subjected to different experimental conditions. Group randomization was hence not relevant.
For animal studies, include a statement about randomization even if no randomization was used.	There was no randomization, "Animals", p18
If the investigator was blinded to the group allocation during the experiment and/or when assessing the outcome, state the extent of blinding. (Give section/paragraph or page #)	Methods section "Animals". No blinding was performed.
For animal studies, include a statement about blinding even if no blinding was done.	Methods section "Animals". No blinding was performed.
For every figure, are statistical tests justified as appropriate?	Methods section "Statistical analyses", Page 30. Due to the hierarchical structure of the data , linear mixed effects statistics on log-transformed data was used. P-values from two-sided test.
Do the data meet the assumptions of the tests (e.g., normal distribution)?	Methods section "Statistical analyses", Page 30
Is there an estimate of variation within each group of data?	Methods section "Statistical analyses", Page 30
Is the variance similar between the groups that are being statistically compared? (Give section/paragraph or page #)	Methods section "Statistical analyses", Page 30

(Continues on following page)

Reagents

Reported in section/paragraph or page #:

To show that antibodies were profiled for use in the system under study (assay and species), provide a citation, catalog number and/or clone number, supplementary information or reference to an antibody validation profile (e.g., Antibodypedia, 1DegreeBio).

Methods, "Immunohistochemistry", page 23. All anitbodies are commercial (see URLs for validation data). Primary antibodies: polyclonal chicken anti-GFP (1:3000, Abcam Cat#ab13970: https://www.abcam.com/gfp-antibody-ab13970.html), rabbit anti-GFP (1:4000, Abcam Cat#ab6556: https://www.abcam.com/gfp-antibody-ab6556.html), mouse anti-NeuN (1:1000, Merck Cat#MAB377), mouse anti-GFAP (1:1000, Merck/Sigma-Aldrich Cat#MAB360: https://www.sigmaaldrich.com/catalog/product/mm/mab377?lang=en&region=NO), rabbit anti-Iba1 (1:1000, Wako Cat#019-19741: https://labchem-wako.fujifilm.com/us/category/01213.html). Secondary antibodies (all diluted 1:200, Jackson Laboratories): Cy5-coupled anti-rabbit (Cat#111-175-144), Cy5-coupled anti-mouse (Cat#115-175-146), FITC-coupled anti-rabbit (Cat#111-095-144) and FITC- coupled anti-chicken (Cat#703-095-155).

7. Cell line identity:

a. Are any cell lines used in this paper listed in the database of commonly misidentified cell lines maintained by ICLAC (also available in NCBI Biosample)?

N/A

b. If yes, include in the Methods section a scientific justification of their use – indicate here on which page (or section and paragraph) the justification can be found.

N/A

c. For each cell line, include in the Methods section a statement that specifies:

- the source of the cell lines

N/A

- have the cell lines been authenticated? If so, by which method?

N/A

- have the cell lines been tested for mycoplasma contamination? In this checklist, indicate on which page (or section and paragraph) the information can be found.

N/A

Animal Models

Reported in section/paragraph or page #:

Report species, strain, sex and age of animals

Methods, "Animals". Male C57BL/6J, *Itpr2*$^{-/-}$ and GLT1-eGFP mice of 8–10 weeks

For experiments involving live vertebrates, include a statement of compliance with ethical regulations and identify the committee(s) approving the experiments.

Methods, section "Animals". All procedures were approved by the Norwegian Food Safety Authority (FOTS 11983).

10. We recommend consulting the ARRIVE guidelines (PLoS Biol. 8(6), e1000412,2010) to ensure that other relevant aspects of animal studies are adequately reported.

Human subjects

Reported in section/paragraph or page #:

11. Identify the committee(s) approving the study protocol.

N/A

12. Include a statement confirming that informed consent was obtained from all subjects.

N/A

13. For publication of patient photos, include a statement confirming that consent to publish was obtained.

N/A

14. Report the clinical trial registration number (at ClinicalTrials.gov or equivalent).

N/A

15. For phase II and III randomized controlled trials, please refer to the CONSORT statement and submit the CONSORT checklist with your submission.

N/A

16. For tumor marker prognostic studies, we recommend that you follow the REMARK reporting guidelines.

N/A

(Continues on following page)

Data Availability

Reported in section/paragraph or page #

7. Provide a Data availability statement in the Methods section under "**Data availability**", which should include, where applicable:
- Accession codes for deposited data
- Other unique identifiers (such as DOIs and hyperlinks for any other datasets)
- At a minimum, a statement confirming that all relevant data are available from the authors
- Formal citations of datasets that are assigned DOIs
- A statement regarding data available with restrictions

See our data availability and data citations policy page for more information.

Data deposition in a public repository is mandatory for:

a. Protein, DNA and RNA sequences
b. Macromolecular structures
c. Crystallographic data for small molecules
d. Microarray data

> Section "Data Availability", page 31:
> "The raw data that support the findings of this study are available from the corresponding author upon reasonable request. The source data underlying Figs. 2c–d, 3b, 4b–c, 5e–g, 6b, e, 7b–e, 8a–d, f, h, 9a, c, and Supplementary Figs. 2a–d, 4b–c, 5e–f, 8g–i, 9, 10c–h, 11, 14a–e, 15 a–c, are provided as Source Data files."
>
> Furthermore a small dataset to illustrate the workflow is available with the Supplementary Software.

Deposition is strongly recommended for many other datasets for which structured public repositories exist; more details on our data policy are available here. We encourage the provision of other source data in supplementary information or in unstructured repositories such as Figshare and Dryad. We encourage publication of Data Descriptors (see Scientific Data) to maximize data reuse

8. If computer code was used to generate results that are central to the paper's conclusions, include a statement in the Methods section under "**Code availability**" to indicate whether and how the code can be accessed. Include version information as necessary and any restrictions on availability.

> Methods, section "Software Availability", page 30. The code and a small dataset i provided as Supplementary Software.
