# Supplementary material for: Astrocytic Ca2+ signaling is reduced during sleep and is involved in the regulation of slow wave sleep
Source: Nat Commun. 2020 Jul 6;11:3240. doi: 10.1038/s41467-020-17062-2 (PMC7338360; doi:10.1038/s41467-020-17062-2)
Supplement: Supplementary file 3 — Supplementary Information [file 41467_2020_17062_MOESM3_ESM.pdf]

Supplementary Information for

Astrocytic Ca^{2+} signaling is reduced during sleep and involved in the regulation of slow wave sleep

Bojarskaite et al.

SUPPLEMENTARY FIGURES

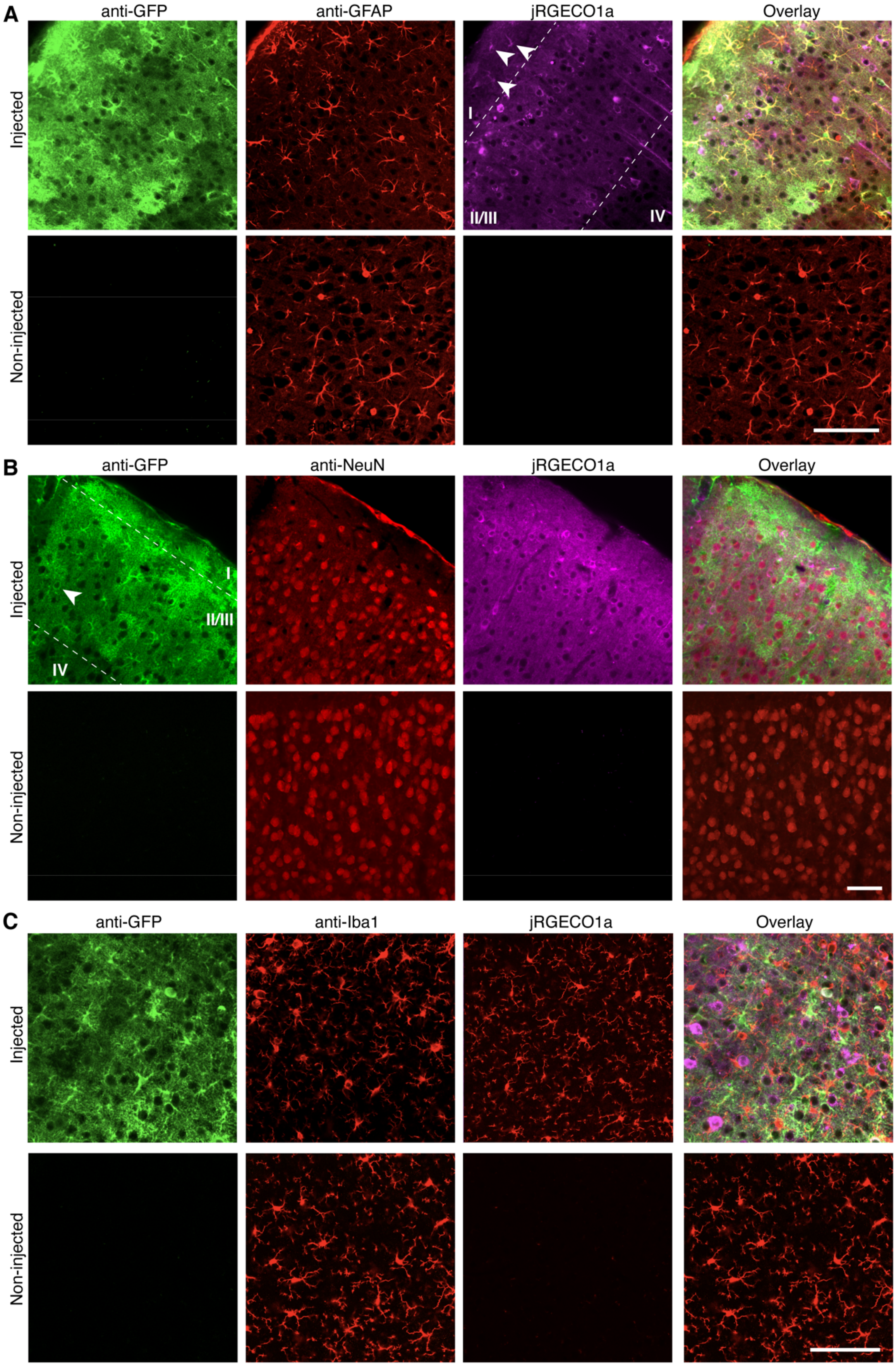
A
anti-GFP
anti-GFAP
jRGECO1a
Overlay
Injected
Non-injected
I
II/III
IV
B
anti-GFP
anti-NeuN
jRGECO1a
Overlay
Injected
Non-injected
I
II/III
IV
C
anti-GFP
anti-Iba1
jRGECO1a
Overlay
Injected
Non-injected

Supplementary Figure 1. Selectivity of the expression of *GFAP*-GCaMP6f in astrocytes and *SYN*-jRGECO1a in neurons in barrel cortex. (A) Immunolabeling of green fluorescent protein (anti-GFP) and glial fibrillary acidic protein (anti-GFAP), as well as jRGECO1a fluorescence demonstrate that astrocytes, and not neurons, were labeled with GCaMP6f. No astrogliosis is observed in the injected hemisphere, as compared to the contralateral non-injected hemisphere. Sparse astrocytic jRGECO1a labeling was noted (white arrowheads), but only in layer I. (B) Immunolabeling with anti-GFP and anti-NeuN, and fluorescence signal of jRGECO1a show that neurons, and not astrocytes, in the virus injection site were labeled with jRGECO1a. Sparse (<1%) neuronal anti-GFP labeling was noted in layer II/III (yellow arrowhead). (C) Immunolabeling with anti-GFP and the microglial marker Iba1 (anti-Iba1), as well as jRGECO1a fluorescence, demonstrate that injection of GCaMP6f and jRGECO1a did not induce microglial activation as compared to the contralateral non-injected hemisphere. All imaging was done in layer II/III. Each immunofluorescence experiment was performed in 3 separate mice. Scale bars 50 μm.

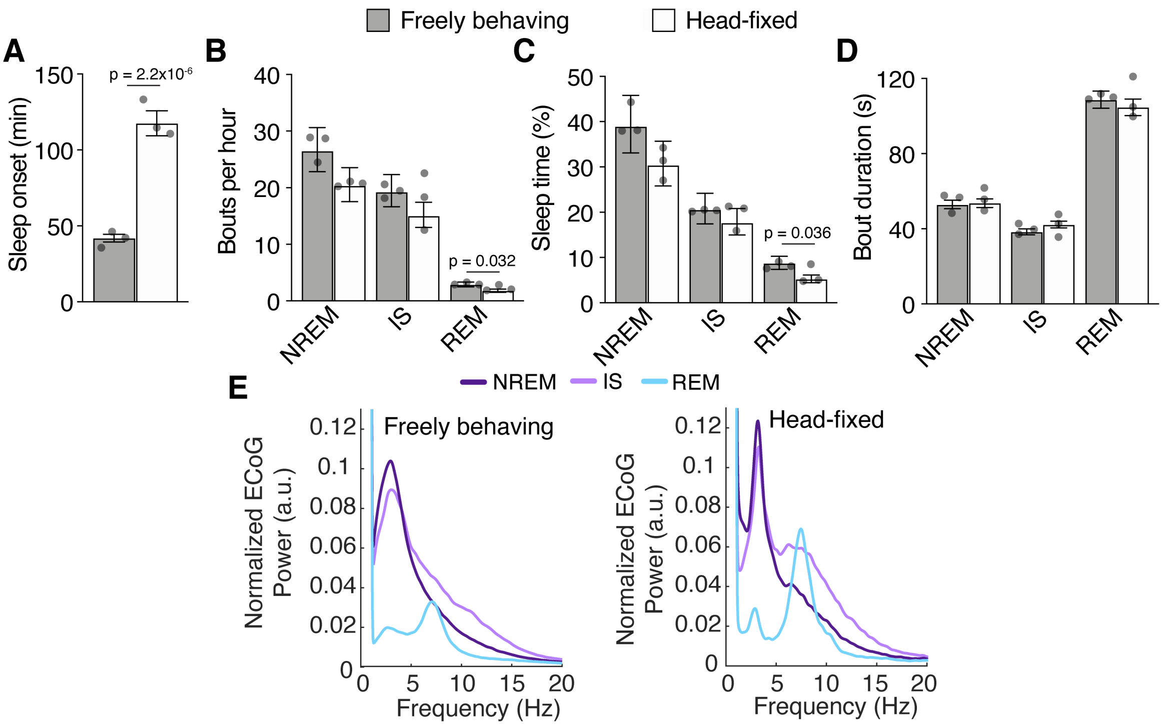

Supplementary Figure 2. Comparison of sleep in head-fixed and freely behaving mice. (A) Sleep onset in freely behaving or head-fixed condition. (B) Average number of NREM, IS and REM sleep bouts per hour of sleep in freely behaving and head-fixed condition. (C) Percentage time spent in NREM, IS and REM sleep out of total sleep time in freely behaving and head-fixed condition. (D) Average NREM, IS and REM sleep bout duration in freely behaving and head-fixed condition. (E) ECoG power spectra of NREM, IS and REM sleep in freely behaving (free) and head-fixed (fixed) condition, normalized to the average total power in the 2–30 Hz frequency range during NREM sleep. Data represented as estimates ± SE and p-values (two-sided test, no adjustment for multiple comparisons) derived from linear regression models for (A–D), mean ± SEM for (E), for freely behaving: n = 3 mice, 5 days, 845 NREM episodes, 673 IS episodes, 102 REM episodes; for head-fixed: n = 3 mice, 5 days, 322 NREM episodes, 279 IS episodes, 35 REM episodes.

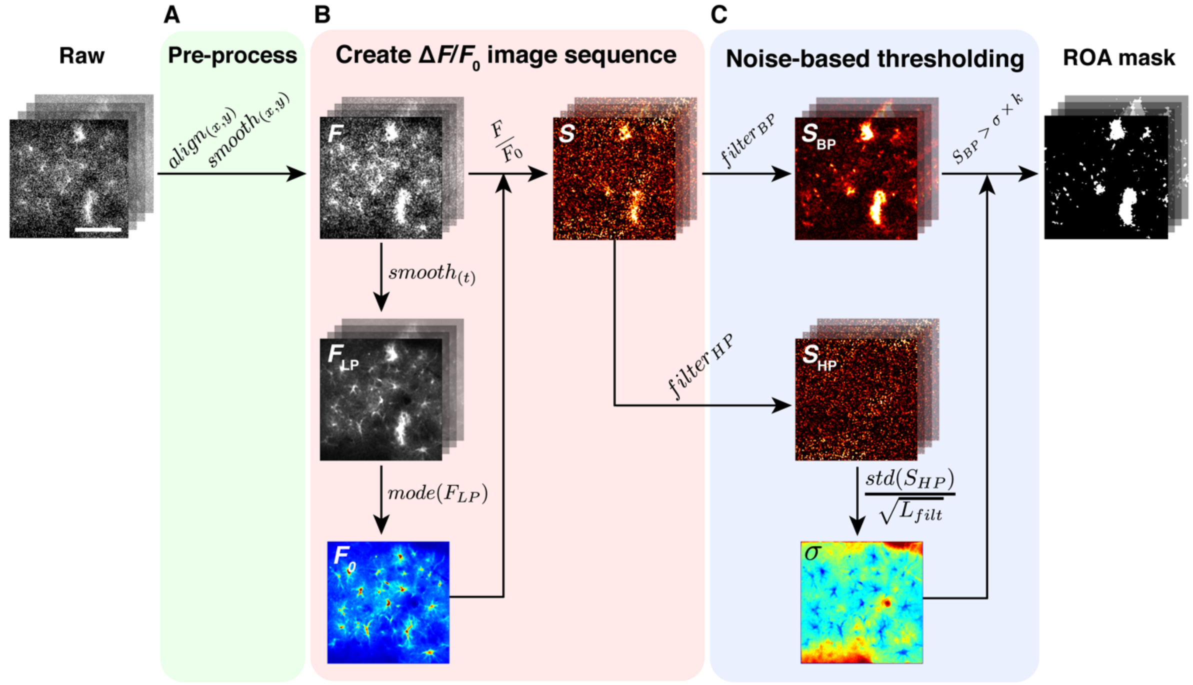

Supplementary Figure 3. Extracting ROAs of astrocyte Ca^{2+} signals from *GFAP*-GCaMP6f fluorescence images. (A) The raw time series is pre-processed via image alignment and short spatial filtering (σ = 2 pixels). (B) A baseline image (F_0) is calculated by first smoothing the time series (moving average width = 1.0 s), resulting in a lowpass filtered time series (F_{LP}), and then subsequently calculating the mode of the pixels. The pre-processed time series *F*, is then divided by the F_0 image, resulting in a $\Delta F/F_0$ time series. (C) The $\Delta F/F_0$ time series is bandpass filtered in time to remove noise and slow drifts in baseline (S_{BP}). A highpass-filtered time series (S_{HP}) is used to estimate the noise and to calculate a standard deviation image (σ). Each pixel in the bandpass filtered time series (S_{BP}) is then thresholded by the corresponding pixel in the σ image multiplied by a factor *k*. Scale bar 50 µm.

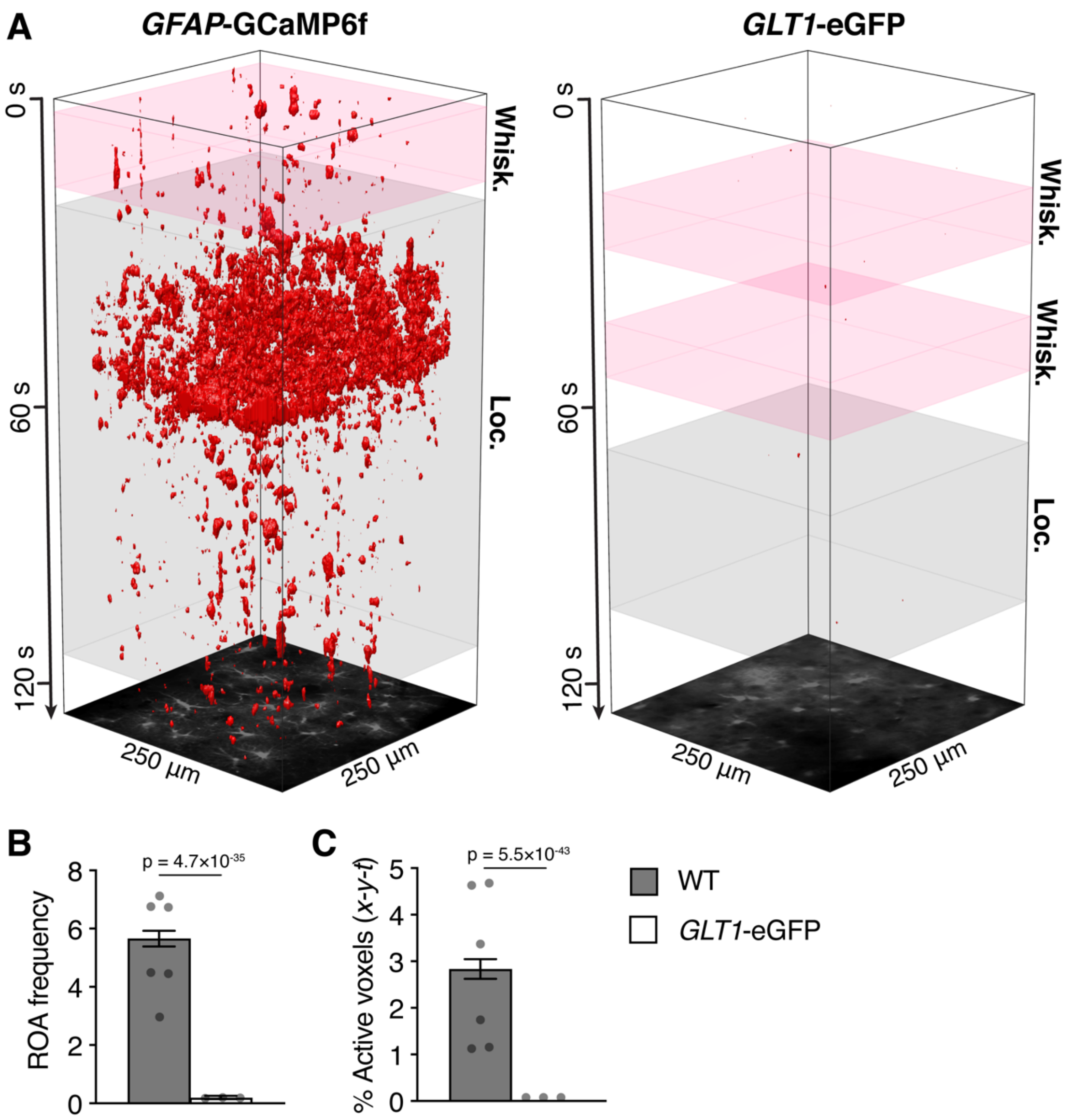

Supplementary Figure 4. Specificity of the ROA algorithm. (A) Representative *x-y-t* rendering of ROAs detected in *GFAP*-GCaMP6f expressing astrocytes (*left*) and in eGFP expressing astrocytes (*right*) in wakefulness during locomotion (Loc.) and whisking (Whisk.). (B and C) ROA frequency (B) expressed as number of ROAs per 100 µm^2 per minute and the percentage of active voxels (*x-y-t*) (C) in the two genotypes. Data represented as estimates ± SE and p-values (two-sided test, no adjustment for multiple comparisons) derived from linear regression models, n = 6 mice, n = 82 trials for WT; n = 3 mice, n = 12 trials for *GLT1*-eGFP.

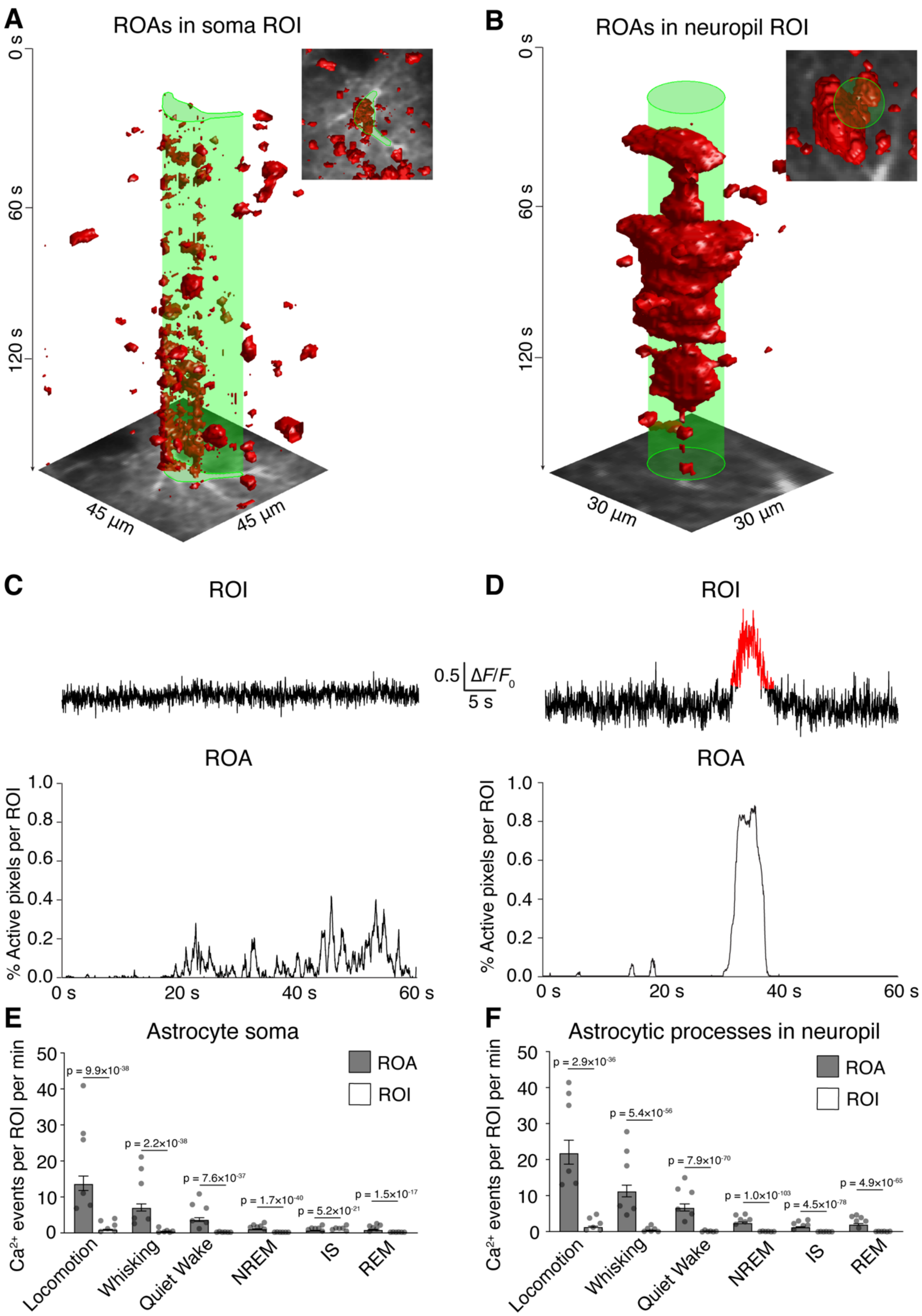

Supplementary Figure 5. Comparison of region-of-interest (ROI) vs. ROA analyses. (A and B) *x-y-t* rendering of ROAs in a field-of-view that contains an astrocyte soma (A) or astrocytic

processes in neuropil (B). (C) Top: $\Delta F/F0$ trace from the astrocyte soma ROI outlined in green. Bottom: percentage active pixels per ROI over time detected with the ROA algorithm. (D) Same as (C), but in neuropil. (E) Ca^{2+} event frequency per astrocyte soma ROI detected by standard ROI analyses compared to the new ROA analysis. (F) Same as (C), but in neuropil. Data represented as estimates ± SE and p-values (two-sided test, no adjustment for multiple comparisons) derived from linear regression models, n = 6 mice, n = 243 trials.

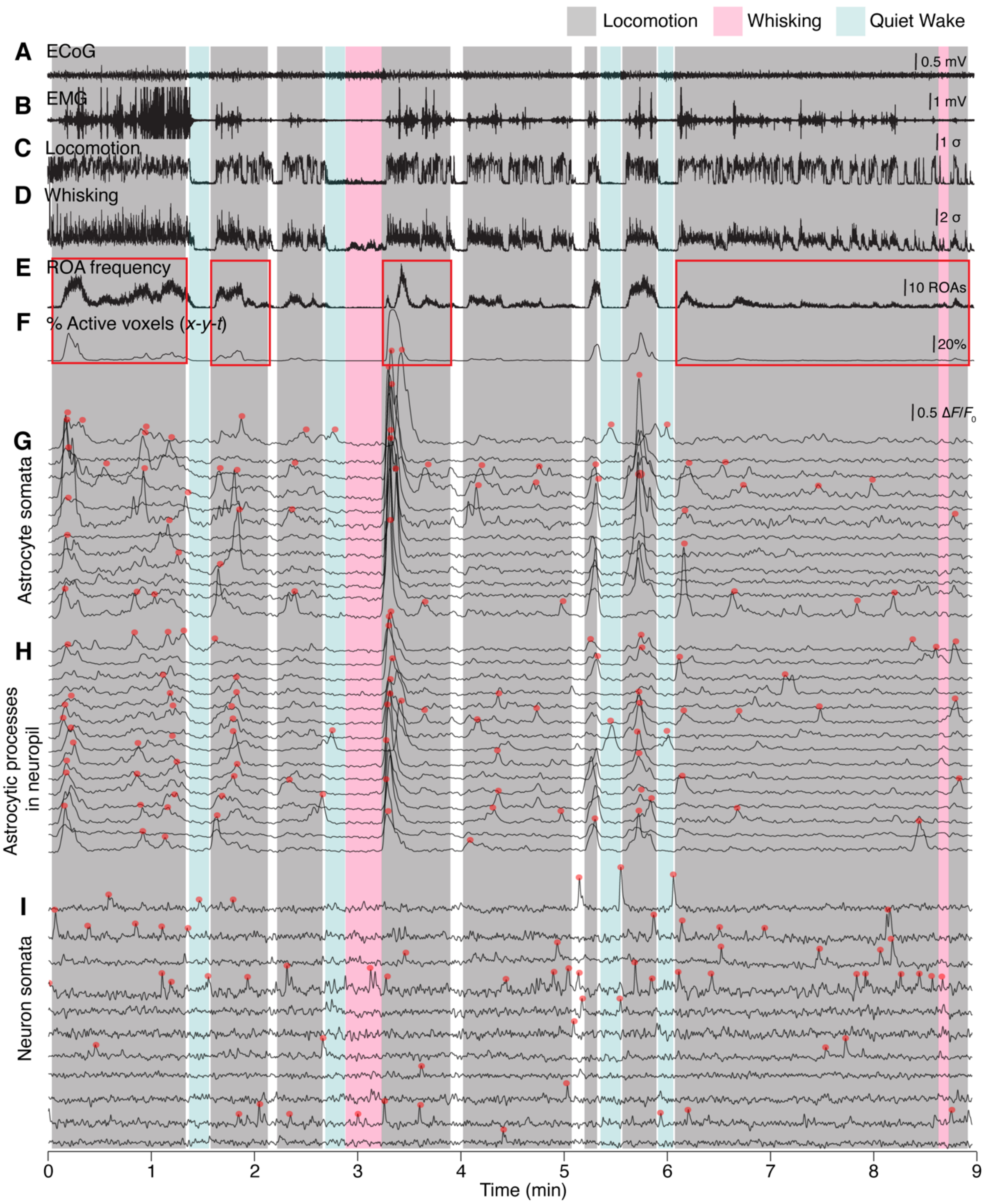

Supplementary Figure 6. A representative wakefulness trial. (A) Bandpass filtered ECoG signal (0.5–30 Hz). (B) EMG signal. (C) Locomotion signal from a ROI placed on the wheel in the infrared-sensitive surveillance camera video. (D) Whisker movement detected from infrared-sensitive surveillance video. (E) ROA frequency expressed as number of ROAs per 100 μm^2 per

minute. (F) Percentage of active voxels (*x*-*y*-*t*). (G) $\Delta F/F_0$ traces from ROIs drawn over astrocyte somata. (H) $\Delta F/F_0$ traces for astrocytic Ca^{2+} signals in neuropil ROIs, including astrocytic processes. (I) $\Delta F/F_0$ traces for ROIs drawn over neuron somata. Red squares illustrate higher astrocytic Ca^{2+} activity related to the beginning of locomotion.

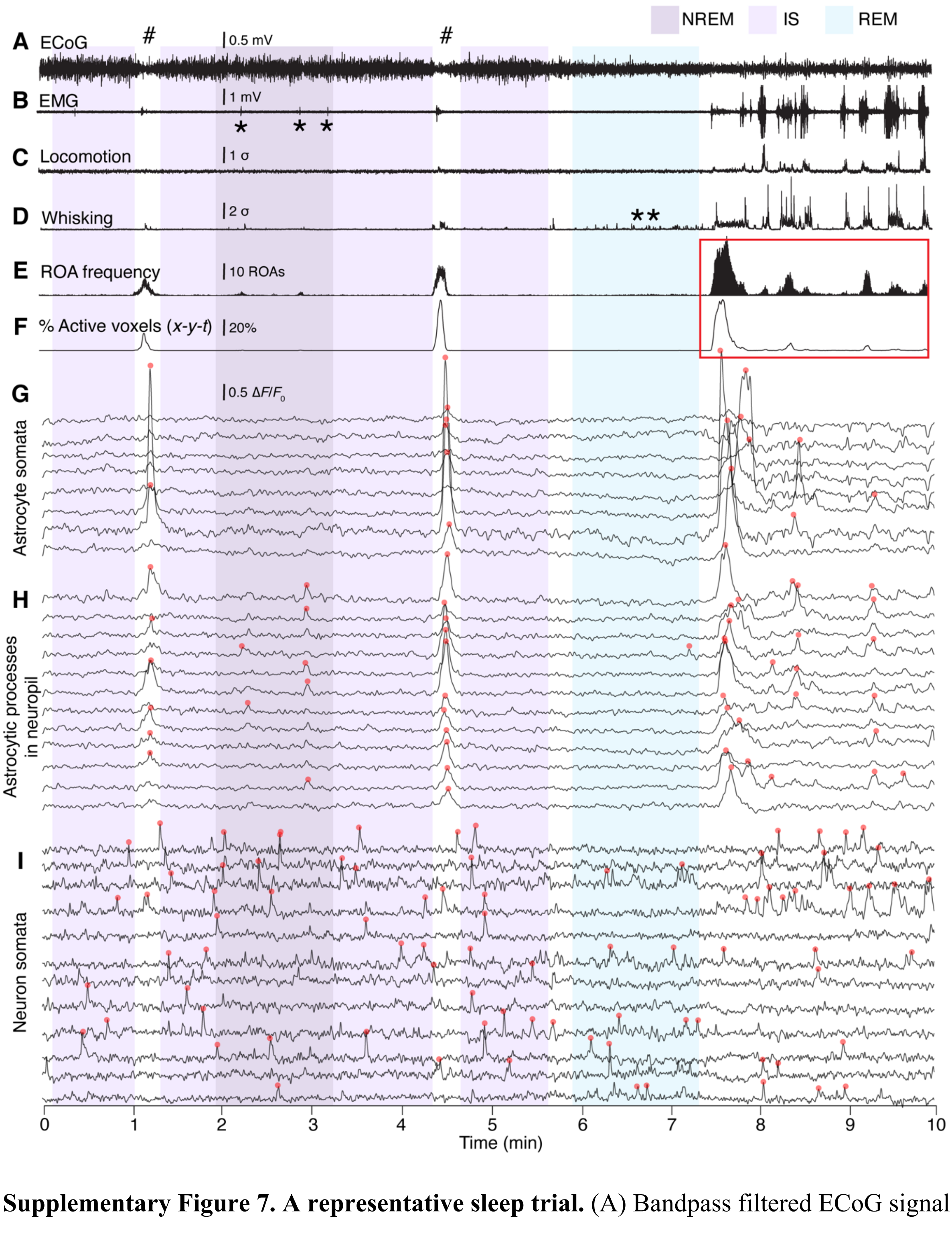

Supplementary Figure 7. A representative sleep trial. (A) Bandpass filtered ECoG signal (0.5–30 Hz). Note an increase in ECoG amplitude during NREM and IS sleep. (B) EMG trace. Note absence of EMG activation during REM sleep. (C) Locomotion signal from a ROI placed on the wheel in the infrared-sensitive surveillance camera video. (D) Whisker movement detected from infrared-sensitive surveillance video. **Note an increase in whisker signal during REM

sleep without EMG activation. (E) ROA frequency expressed as number of ROAs per 100 μm^2 per minute. (F) Percentage of active voxels (*x*-*y*-*t*). (G) $\Delta F/F_0$ traces from ROIs drawn over astrocyte somata. (H) $\Delta F/F_0$ traces for astrocytic Ca^{2+} signals in neuropil ROIs, including astrocytic processes. (I) $\Delta F/F_0$ traces for ROIs drawn over neuron somata. Red squares illustrate increased astrocytic Ca^{2+} activity during an awakening. Single asterisk * indicates microarousals. Symbol # indicates short awakenings that are excluded from SWS.

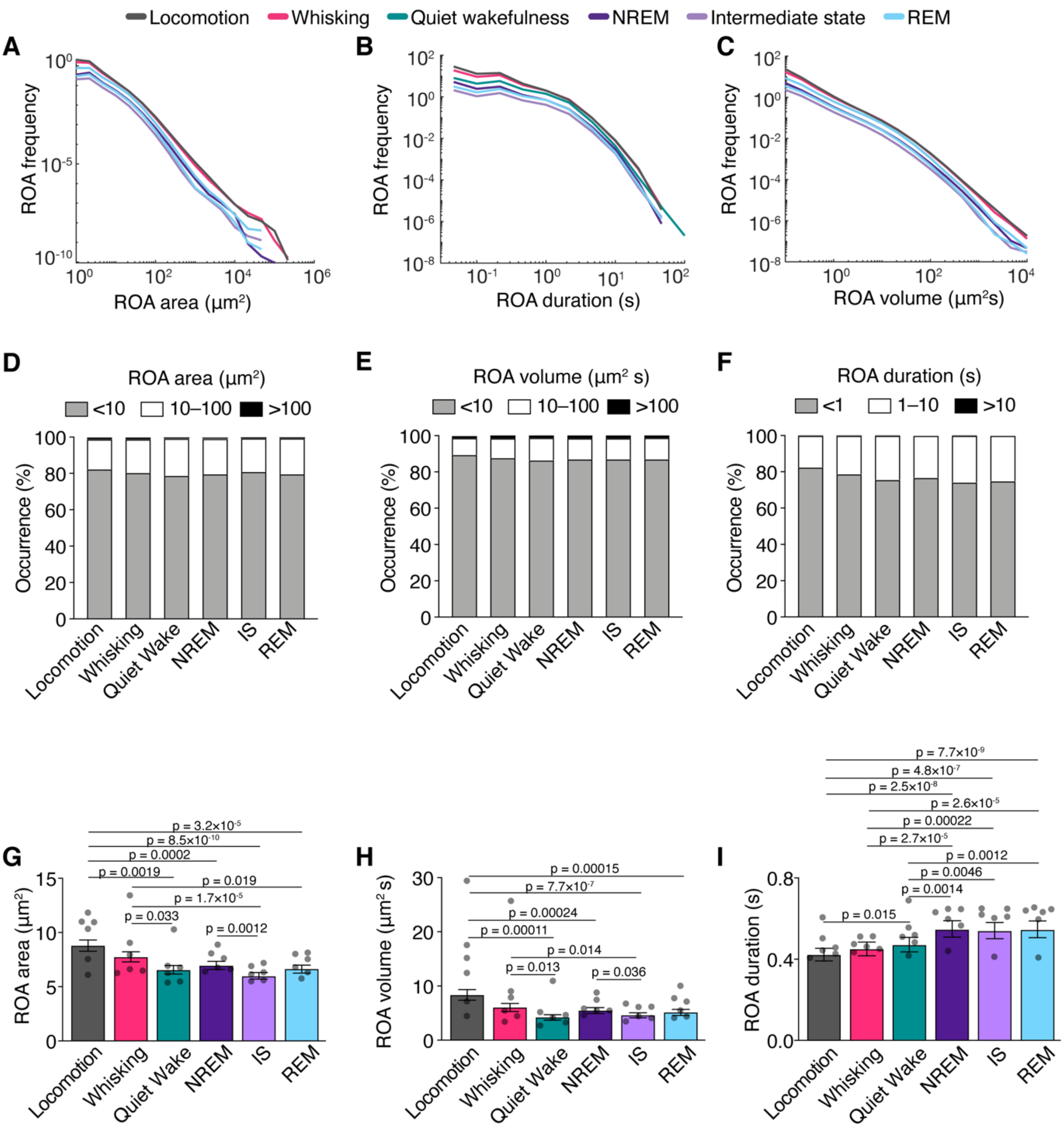

Supplementary Figure 8. **Spatiotemporal characteristics of ROAs.** (A to C) The relationship between ROA frequency (y-axis) and ROA area (A), ROA duration (B) and ROA 3D (*x*-*y*-*t*) volume (C) across sleep-wake states. (D) Occurrence of small (<10 µm^2), medium (10–100 µm^2) and large (>100 µm^2) area ROAs across sleep-wake states. (E) Occurrence of small (<10 µm^2 s), medium (10–100 µm^2 s) and large (>100 µm^2 s) volume ROAs across sleep-wake states. (F) Occurrence of short (<1 s), medium (1–10 s) and long (>10 s) duration ROAs across sleep-wake

states. (G to I) Mean ROA area (G), mean ROA volume (H), and mean ROA duration (I) across sleep-wake states. Data represented as estimates ± SE and p-values (two-sided test, no adjustment for multiple comparisons) derived from linear mixed effects models statistics, n = 6 mice, n = 278 trials, n = 2 318 567 ROAs. For details on statistical analyses, see Methods.

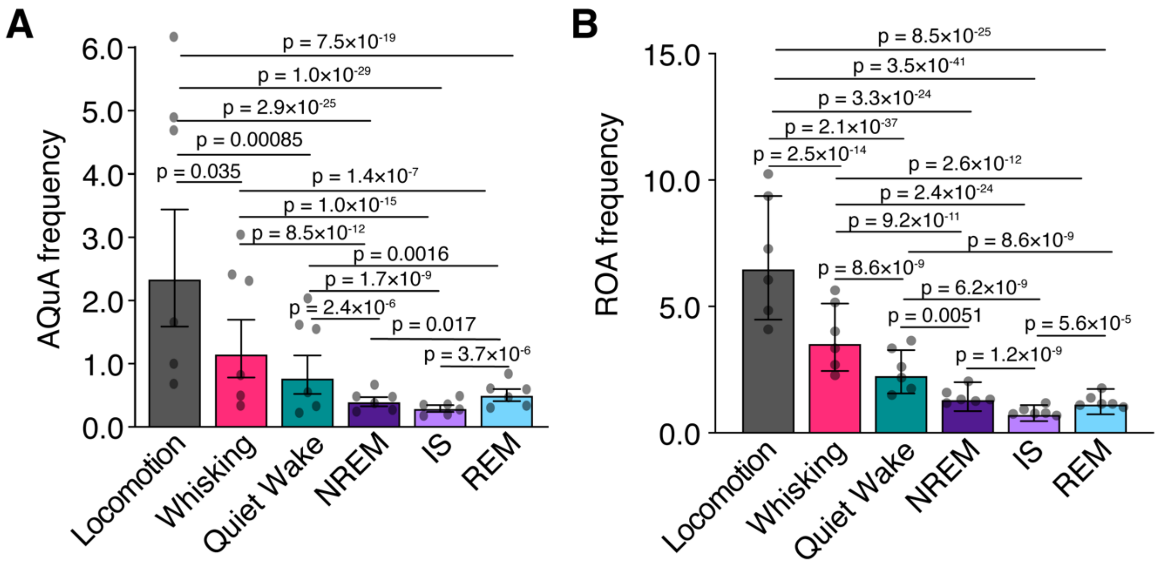

Supplementary Figure 9. Ca^{2+} signaling in astrocytes across sleep-wake states analyzed by AQuA software. (A and B) Ca^{2+} signaling in astrocytes across sleep-wake states analyzed by AQuA (A) and ROA (B) methods. Data represented as estimates ± SE and p-values (two-sided test, no adjustment for multiple comparisons) derived from linear mixed effects models statistics, n = 6 mice, 278 trials. For details on statistical analyses, see Methods.

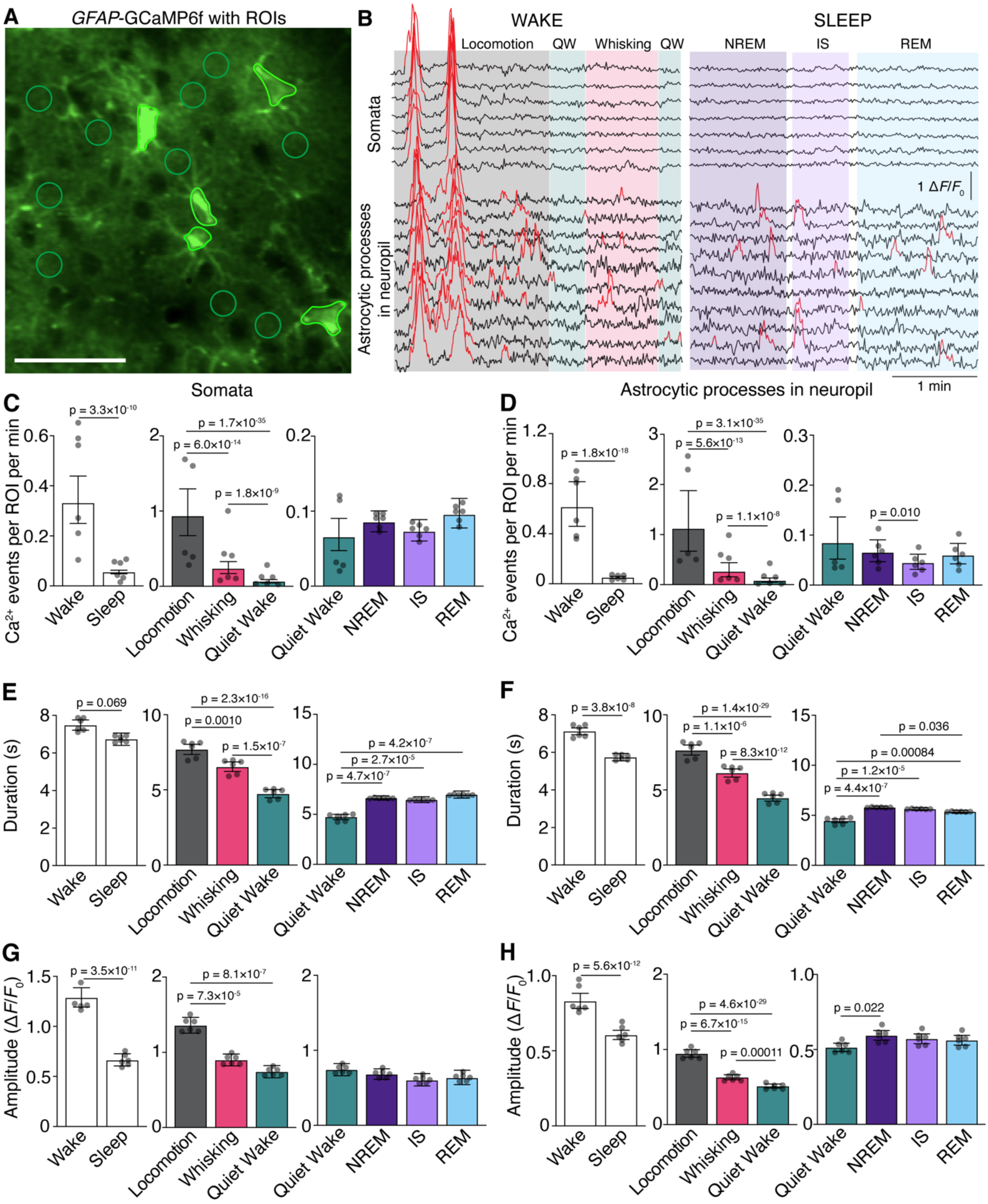

Supplementary Figure 10. Astrocytic Ca^{2+} events across sleep-wake states from hand-drawn ROIs. (A) Representative average image of *GFAP*-GCaMP6f fluorescence in astrocytes, and ROIs over astrocyte somata and neuropil. In total, 284 trials from 6 mice were manually

segmented with ROIs. Scale bar 100 μm. (B) Example $\Delta F/F_0$ Ca^{2+} traces from astrocyte somata and neuropil ROIs across sleep-wake states. Detected Ca^{2+} peaks are indicated in red. Traces smoothed by a 30-frame moving median filter. (C and D) Frequency of Ca^{2+} signals in astrocyte somata (C) and neuropil (D) during overall wakefulness and overall sleep (*left*), during locomotion, whisking and quiet wakefulness (*middle*), and during NREM, IS and REM sleep compared to quiet wakefulness (*right*). (E and F) Duration of Ca^{2+} signals in astrocyte somata (E) and neuropil (F) during overall wakefulness and overall sleep (*left*), during locomotion, whisking and quiet wakefulness (*middle*), and during NREM, IS and REM sleep compared to quiet wakefulness (*right*). (G and H) Same as in (E and F), but for amplitude of Ca^{2+} signals instead of duration. Data represented as estimates ± SE and p-values (two-sided test, no adjustment for multiple comparisons) derived from linear mixed effects models statistics, n = 6 mice, 284 trials for (C, E, G); 278 trials for (D, F, H). For details on statistical analyses, see Methods.

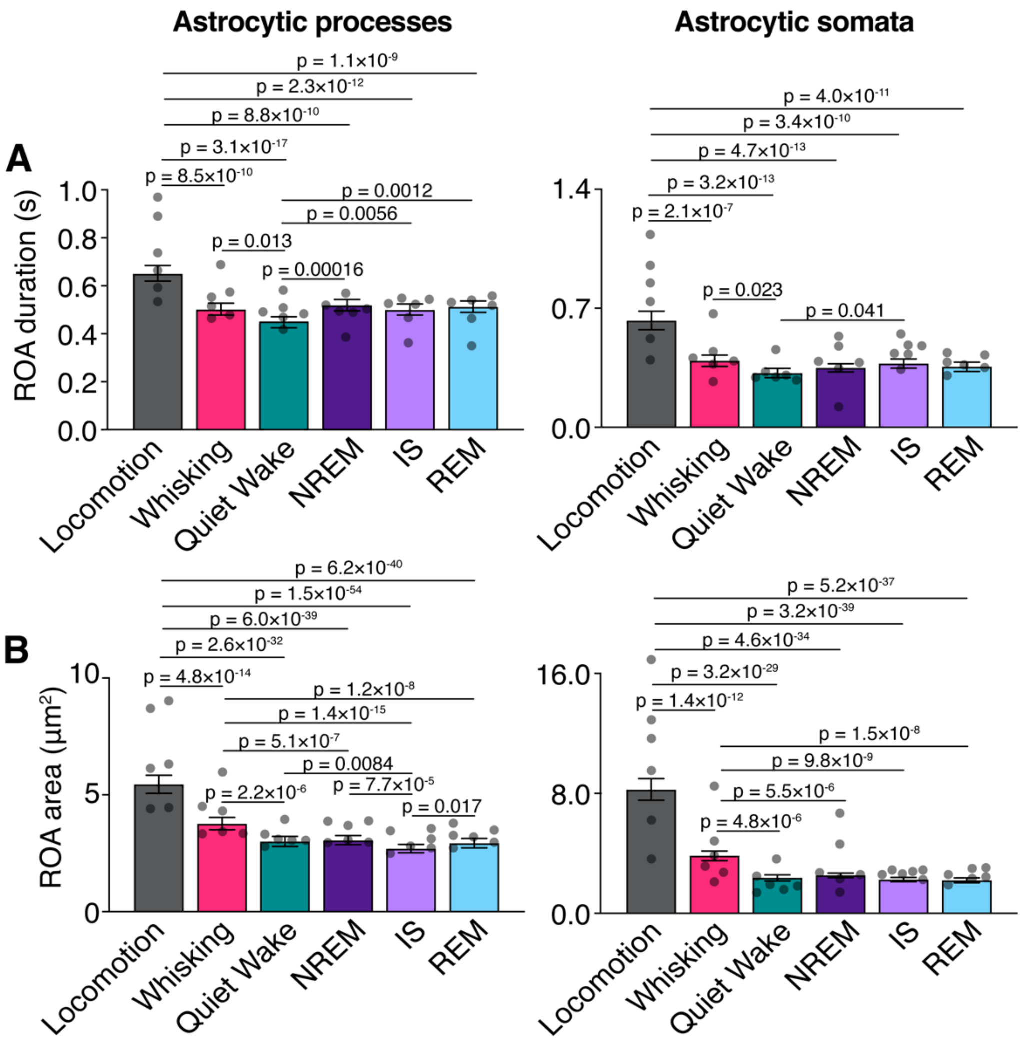

Supplementary Figure 11. Spatiotemporal characteristics of ROAs in astrocytic processes and astrocytic somata. (A) Mean ROA duration in astrocytic processes (*left*) and astrocytic somata (*right*) across sleep-wake states. (B) same as (A) but for mean ROA area. Data represented as estimates ± SE and p-values (two-sided test, no adjustment for multiple comparisons) derived from linear mixed effects models statistics, n = 6 mice, 283 trials. For details on statistical analyses, see Methods.

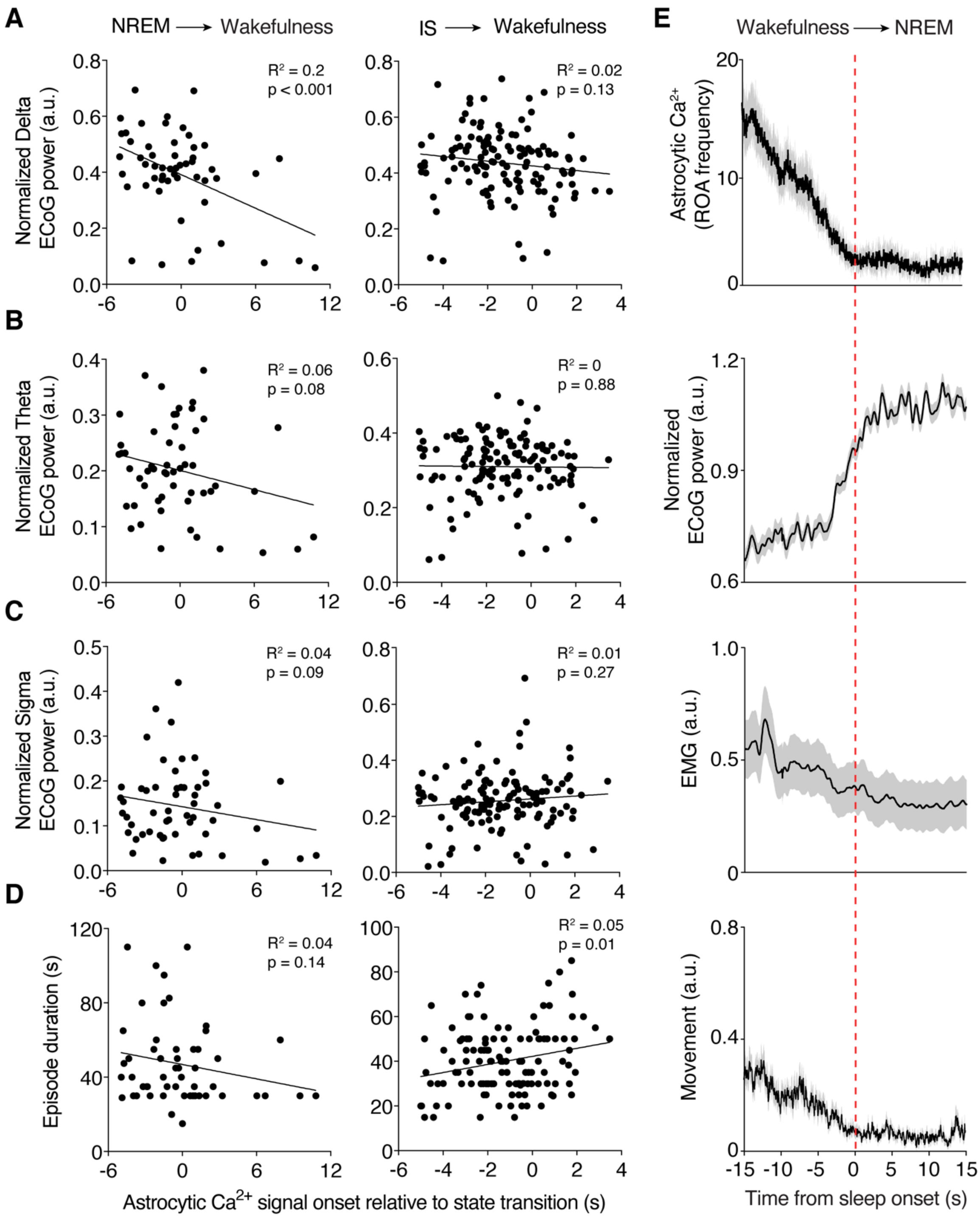

Supplementary Figure 12. Astrocytic Ca^{2+} signaling at sleep-wake state transitions. (A-D) Correlation between astrocytic Ca^{2+} signal onset and ECoG power in delta (A), theta (B) and sigma (C) frequency bands, and NREM or IS sleep episode mean duration (D) in relation to state transition from NREM sleep (*left*) or IS sleep (*right*) to wakefulness. n = 6 mice, 59 NREM sleep to wakefulness transitions, 140 IS sleep to wakefulness transitions. (E) Mean time-course of (*top*

to bottom) astrocytic Ca^{2+} signals expressed as a number of ROAs per 100 μm^2 per minute, and traces of total ECoG power 0.5–30 Hz, EMG and mouse movement during transitions from wakefulness to NREM sleep. ECoG power in A–E was normalized to the average total power in the 0.5–30Hz frequency range during NREM sleep. Traces represent the mean ± SEM, n = 6 mice, 429 transitions. P-values obtained from simple linear regression performed in Graphpad Prism.

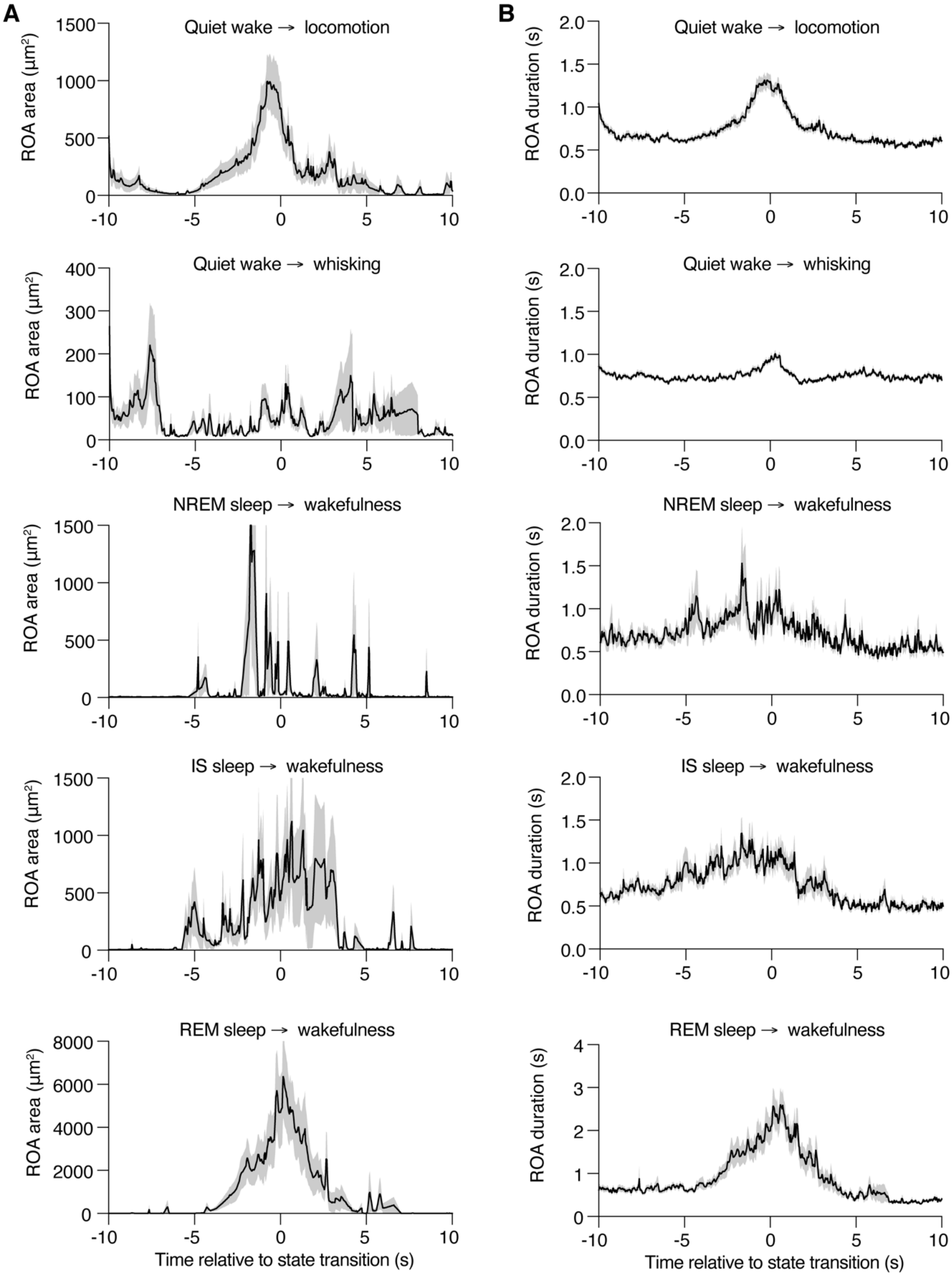

Supplementary Figure 13. ROA area and duration at brain state transitions. (A) Mean ROA area during transitions (top to bottom) from quiet wakefulness to locomotion (n = 199 transitions), quiet wakefulness to whisking (n = 370 transitions), from NREM sleep to wakefulness (n = 59 transitions), from IS sleep to wakefulness (n = 140 transitions) and from

REM sleep to wakefulness (n = 87 transitions). (B) same as (A) but for mean ROA duration. Traces represent the mean ± SEM, n = 6 mice.

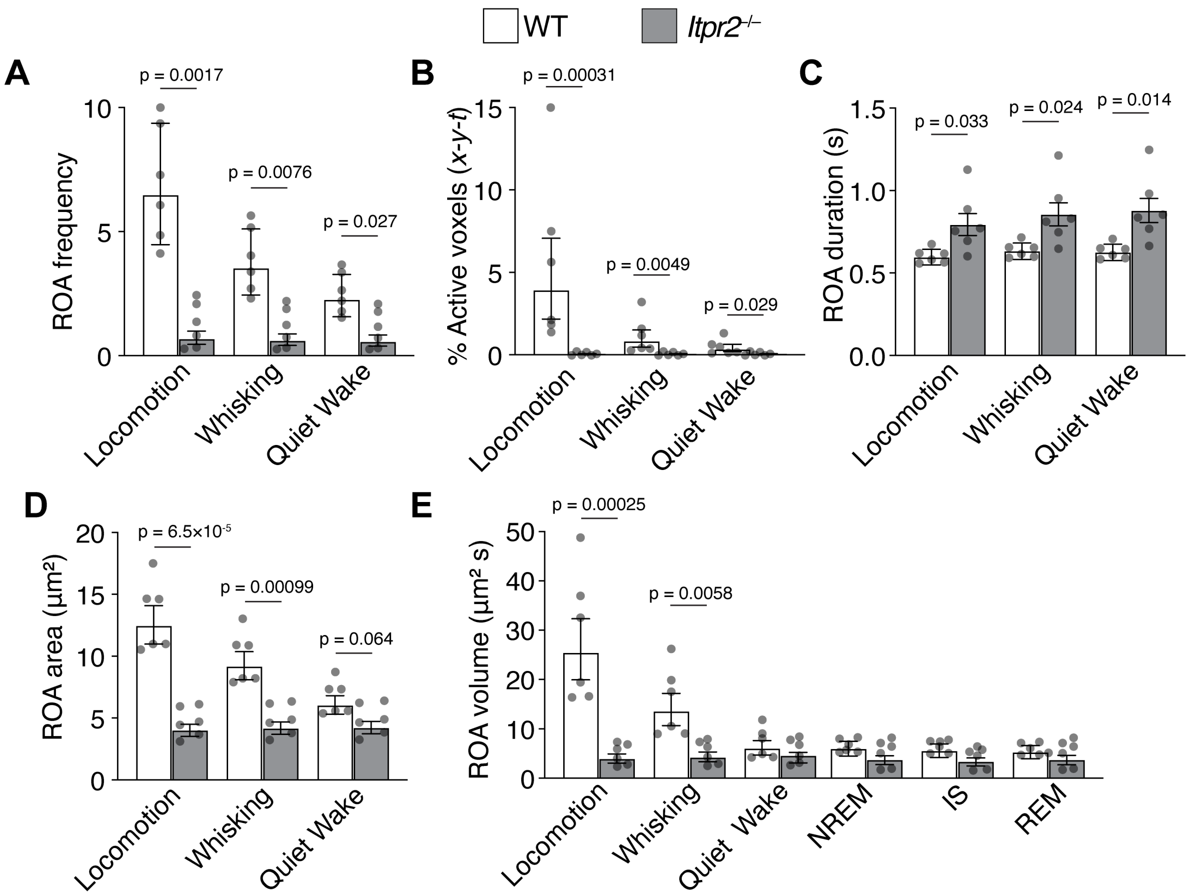

Supplementary Figure 14. Astrocytic and neuronal Ca^{2+} signaling in *Itrp2*$^{-/-}$mice during sleep-wake states. (A to E) Mean ROA frequency expressed as number of ROAs per 100 µm^2 per minute (A), the percentage of active voxels (*x-y-t*) (B), ROA duration (s) (C), ROA area (µm^2) (D) during locomotion, whisking and quiet wakefulness, and ROA volume (µm^2 s) (E) across sleep-wake states in WT and *Itpr2*$^{-/-}$ mice. For WT mice: n = 6 mice, 82 trials; for *Itpr2*$^{-/-}$ mice: n = 6 mice, 96 trials. For WT mice: n = 6 mice, 25 trials; for *Itpr2*$^{-/-}$ mice: n = 5 mice, 70 trials. Data represented as estimates ± SE and p-values (two-sided test, no adjustment for multiple comparisons) derived from linear mixed effects models statistics. For details on statistical analyses, see Methods.

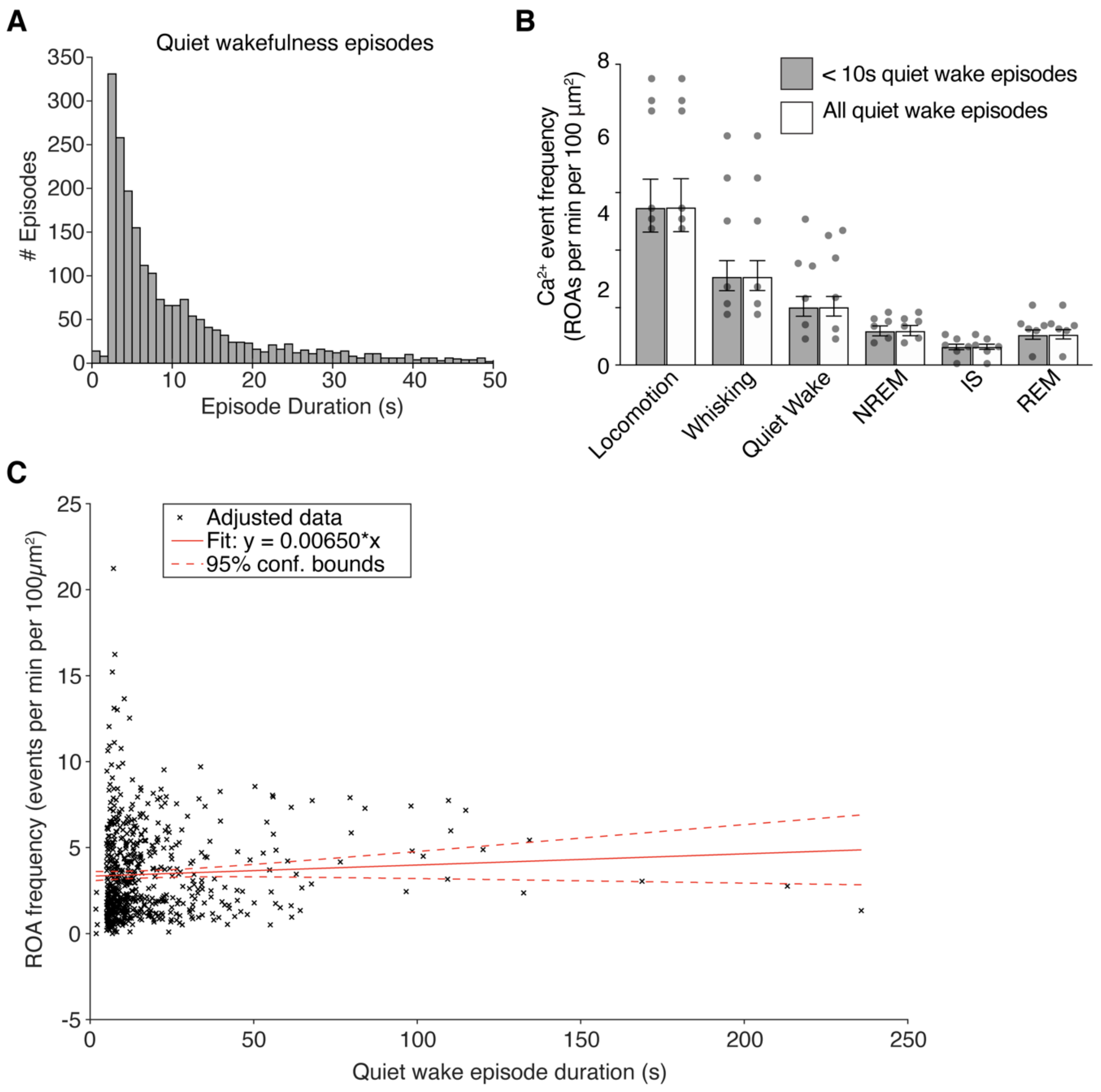

Supplementary Figure 15. Quiet wakefulness episode characteristics. (A) Distribution of the durations of quiet wakefulness episodes is skewed towards short episodes (>90% of bouts are <10 s, n = 12 mice, 174 trials, 2161 quiet wake bouts). (B) Astrocytic Ca^{2+} event frequency across sleep-wake states including all quiet wakefulness episodes compared to including only <10 s quiet wakefulness episodes. Data represented as estimates ± SE and p-values (two-sided test, no adjustment for multiple comparisons) derived from linear mixed effects models statistics, n = 6 mice, 276 trials. (C) No correlation is observed between quiet wakefulness episode duration and astrocytic Ca^{2+} event frequency. n = 6 mice, 78 trials, 615 datapoints.
