# Supplementary material for: Astrocytic Ca2+ signaling is reduced during sleep and is involved in the regulation of slow wave sleep
Source: Nat Commun. 2020 Jul 6;11:3240. doi: 10.1038/s41467-020-17062-2 (PMC7338360; doi:10.1038/s41467-020-17062-2)
Supplement: Supplementary file 8 — Supplementary Software [file 41467_2020_17062_MOESM8_ESM.zip › sleep_project_demo/readme.rtf]

1. System Requirements
A computer with 16 GB RAM and Matlab 2018a or later. 
Software has been tested on Matlab 2018a. 

2. Installation guide: Short Explanation
	1. Extract Supplementary Software
	2. Install Matlab. 
	3. Run Matlab.
	4. Find “sleep_project_demo” folder in Matlab, right click and press “Add to Path” -> “Selected Folder and Subfolders”.

2. Installation guide: Detailed Explanation
First make sure necessary folders are available. These are: 
	- sleep_project_demo
	- demo_data
“sleep_project_demo” contains our custom code. 
“demo_data” contains a small excerpt of our dataset. The file is around 1GB. 

To run our code Matlab first has to be installed. Matlab is a proprietary computing environment and programming language that requires a license to run. Installation instructions are available through Matlab’s own website, https://se.mathworks.com/help/install/. Matlab is a large program (around 30 GB, depending on which tools are included) and installation often lasts several hours. 

Uncompress the “sleep_project_demo.zip” file and locate the contents. The content should be a folder named “sleep_project_demo” and contains several subfolders. Also uncompress “demo_data.zip”, this data will be loaded when the demonstration program is running. 

Once Matlab is installed a directory with the name “MATLAB” is created inside the “Documents” directory. Move the directory “sleep_project_demo” into the “MATLAB” folder. 

Run Matlab and look in the left panel, named “Current Folder”. Inside this panel should be the “sleep_project_demo” folder. If somehow the left panel does not display the “sleep_project_demo” folder, use the navigation tools above the panel to locate the “sleep_project_demo” folder.

Right click the “sleep_project_demo” folder, hover over “Add to Path” and press “Selected Folder and Subfolder”. The code is now ready to be used. 

3. Demo
	1. Locate and open +demo/main.m
	2. Run the first section, with the title “Load dataset”. To run a section place the cursor inside a section and press command + enter  (ctrl + enter on windows).  
	3. A window will appear, select the demo_dataset folder. When the correct folder is selected the dataset will be loaded and the output should look something like this:

BEGONIA_VERBOSE log level set to 2
[21:14:37] : Looking for tseries
[21:14:37] : Discovering directories
[21:14:37] : Scanning folder (9/9)
[21:14:37] : Found 1 objects
[21:14:37] : Looking for trials
[21:14:37] : Discovering directories
[21:14:37] : Scanning folder (9/9)
[21:14:37] : Found 1 trials
[21:14:37] : Associating objects by time info
[21:14:37] : Number of associated elements    : 1
[21:14:37] : Mean time offset                 : 00:00:05
[21:14:37] : Std time offset                  : 00:00:00
[21:14:37] : Min time offset                  : 00:00:05
[21:14:37] : Max time offset                  : 00:00:05

	4. Run the second section, with the title “Processing workflow GUI”. This will open a window with a table to the left and several buttons on the right. The rightmost buttons represent our processing steps for calculating and plotting ROA. Each button loads some data, processes it and saves the processed data again. The processed data can then be used by subsequent processing steps (buttons). Only 3 of the buttons have visual outputs, these are “Configure Camera”, “ROA Ignore Tool” and “ROA 3D Tool”. The table to the left indicate which data is in the trials of the dataset. The demo dataset only contain one trial, which is why there is only one row. The columns represent keywords that are used to save or retrieve data. To run a process select a cell in the table and press a button on the right side. We have pre-selected which data is visible in the table. Each of the columns contain the data that is saved under the column name. By selecting a cell in the table and pressing the “open” button the raw data will be displayed in the terminal. Many of the cells have data does not look good when displayed in the terminal. The last variable named “roa_per_episode” is a table and displays easily when pressing “open”. 

Technical detail: Each of the buttons are connected to a single processing function, these can be seen by opening the “demo.gui.demo_gui” file. The processing functions have access to the dataset via trial objects. Trial objects are something we have created to have an easy way to load and save data. Data can be saved to the trials by supplying a keyword along with some data, or reversely data can be loaded by entering a keyword to retrieve previously saved data.

	5. Press each of the rightmost buttons to process data. Below is a short description of each of the processing steps / buttons. Many of the steps do not have visual outputs and simply process data from the previous step. The processing has already been completed and is saved in the data, it is therefor possible to skip steps in this demonstration. The maybe most visually interesting processing step is the ROA Ignore tool, which can displays the ROAs together with the calcium recording. 

Configure Camera: Loads the camera file and enables the user to mark areas that cover the wheel and whisker. The information about which areas are marked and what that area represents is stored under the keywords “video_region_names” and “video_region_mask”. 

Run Camera: Loads the camera file, “video_region_names” and “video_region_mask” to extract the marked areas of the camera into time series traces. The time series traces are then stored to a new keyword “camera_regions”. 

Load Manual Scoring: Loads a manually created excel file(s) with information about the timing of the sleep phases. The information in the excel document is used to create a categorical vector which indicates which sleep state the mouse is in with a sampling rate of 30 Hz. As the mouse in the demo dataset is awake the vector is filled with the state, aptly named, “pre_sleep”. The vector is saved under the keyword “sleep_stages”.

Run States : Loads the “sleep_stages” vector, along with the activity traces from the camera (“camera_regions”) and defines the behavior states shown in the article. For each continuous episode a row is added to a table with information about the state, start point and end point. The table is stored under the variable “state_episodes”.

Calculate ROA Mask: Loads the calcium recording and applies the ROA algorithm as explained in the methods. The 3D binary ROA mask of the video is stored under the keyword “highpass_roa_mask”. 

Threshold ROA Mask: Loads the 3D binary ROA mask and excludes events smaller than 0.85 um^2. This was to standardize the smallest events as some trials had different zoom levels. The value of 0.85 um^2 represents the size of one pixel in the most zoomed out trials. The thresholded 3D binary ROA mask is then stored under the keyword “highpass_thresh_roa_mask”.

ROA Ignore Tool: Opens a GUI displaying the calcium recording and enables the user to mark areas that should not be included when calculating measurements from the ROA. By pressing the Load ROA button an overlay of the previously processed ROAs will be displayed. Clicking and dragging the mouse on the GUI marks the surrounding area with a blue tint. The ignored areas are saved under the keyword “roa_ignore_mask”. 

ROA Traces: Loads “highpass_thresh_roa_mask” and “roa_ignore_mask” to create traces of the number of ROAs that start at every frame, the % of active pixels at every frame as well as a table with each ROA with information about their duration, size and volume. This information is stored under “highpass_thresh_density_trace”, “highpass_thresh_roa_frequency_trace” and “highpass_thresh_roa_table”.

ROA per Episodes: Loads the “highpass_thresh_roa_frequency_trace” and “highpass_thresh_density_trace” together with “state_episodes” (the information about each behavior episode) to calculate the frequency and % active pixels in each episode. The results are gathered in a table under the variable “roa_per_episode”. 

ROA 3D Tool: Opens a GUI that can create outlines of the ROAs or plot them in 3D. Write down the frames which should be included in the plots and press one of the buttons. 

	6. Run the third section, with the title “Load a data table”. When the last processing step has been completed, “roa_per_episode” in this case, we like to extract all the data from every trial (only one trial is present in demo_data) into a table that is used for statistics and plots later. 

	7. We have used the same workflow to analyse most of our data. More of the processing GUIs can be found under “+alyssum_v2/+guis”. As an example of this run the last section, with the title “ROI workflow GUI”. This will show a GUI with buttons for different processing steps for the ROIs. The first step is marking the ROIs, clicking the “ROI Manager” button will open the ROI marking tool. 

Instructions for use
To run the original software the whole dataset (20 TB) must be available. The code is initiated with the TrialManager, as in the demo code, located at “alyssym_v2.TrialManager”. The TrialManager is passed to GUI functions, located at “alyssum_v2.guis”.

All loading of data from the raw file formats into Matlab are a done using the same workflow as in the demo_gui, the only exception is EEG and EMG which was loaded with our older system (+alyssum). The older systems is started from alyssum.main.load_trial_manager which locate trials in the dataset. Those trials are then passed to functions inside alyssum.trial_functions to create various tables. Those tables were then loaded with a TableProcessor, which handled the rest of the processing and plotting procedures. The functions alyssum.main.load_trial_manager and alyssum.main.load_table_processor is sentral to the old system. 

Descriptions of the folders:
	+alyssum : 
Older project specific code.  

	+alyssum_v2 : 
Project specific code. 

	+begonia : 
Shared code for our group associated with analysis (including ROA). 

	+xylobium : 
Shared code for our group containing GUIs. 

	+yucca : 
Shared code for our group associated with our data recording equipment (but not calcium recordings, that is in begonia).

	+calendula : 
Code associated with the ROI marking tool. 

	roimanager_lite : 
The ROI marking tool. 

	export_fig : 
3rd party functions for plotting matlab figures. 

	gui_layout_toolbox: 
3rd party functions for creating GUIs.

(Added after second submission)
	+dogbane
Code associated with GCaMP data but newer analysis. 

	+dogbane_2
Code associated with data comparing sleep in head fixed vs. freely sleeping.
