# Supplementary material for: Astrocytic Ca2+ signaling is reduced during sleep and is involved in the regulation of slow wave sleep
Source: Nat Commun. 2020 Jul 6;11:3240. doi: 10.1038/s41467-020-17062-2 (PMC7338360; doi:10.1038/s41467-020-17062-2)
Supplement: Supplementary file 8 — Supplementary Software [file 41467_2020_17062_MOESM8_ESM.zip › sleep_project_demo/gui_layout_toolbox/layoutdoc/Function_reference4_3.html]

layoutRoot


|  |  |
| --- | --- |
| **Function\_reference** |  |

  

## 4.3: layoutRoot

returns the folder containing the GUI layout toolbox

`folder` `= layoutRoot(` `)`
:   returns the full path to the folder containing the GUI Layout Toolbox.

### For example:

```
folder = layoutRoot()
```

`folder = 'C:\tools\glt2'`

  

|  |  |  |  |  |
| --- | --- | --- | --- | --- |
|  | uix.tracking | [Top] | Front page |  |

© 2018 The MathWorks Ltd
• Terms of Use
• Patents
• Trademarks
>
