# Supplementary material for: Astrocytic Ca2+ signaling is reduced during sleep and is involved in the regulation of slow wave sleep
Source: Nat Commun. 2020 Jul 6;11:3240. doi: 10.1038/s41467-020-17062-2 (PMC7338360; doi:10.1038/s41467-020-17062-2)
Supplement: Supplementary file 8 — Supplementary Software [file 41467_2020_17062_MOESM8_ESM.zip › sleep_project_demo/gui_layout_toolbox/layoutdoc/User_guide7_2.html]

createInterface


|  |  |
| --- | --- |
| **User\_guide** |  |

  

## 7.2: createInterface

The interface creation is handled in the `createInterface`
subfunction. This has two distinct sections: menu building and widget arrangement.
The menus are built using the standard MATLAB menu building command `uimenu`,
so let's concentrate on the widget arrangement.

The top-level layout is a horizontal arrangement, placing the controls
to the left of the main plot. We make the layout draggable by using the
"flex" variant of HBox, and put a panel in each side. Note that setting the
"HelpFcn" for the view panel adds a small "?" icon for bringing up help. See
here for more details.

```
% Add the contents
mainLayout = uix.HBoxFlex( 'Parent', gui.Window, 'Spacing', 3 );

% Create the panels
controlPanel = uix.BoxPanel( ...
   'Parent', mainLayout, ...
   'Title', 'Select a demo:' );
gui.ViewPanel = uix.BoxPanel( ...
   'Parent', mainLayout, ...
   'Title', 'Viewing: ???', ...
   'HelpFcn', @onDemoHelp );

% Adjust the main layout  

set( mainLayout, 'Widths', [-1,-2]  );
```

The controls panel is filled with a vertical layout containing the listbox
and a button. Note the callbacks that are specified for both the list and button. These
both call further subfunctions that are able to access the common "data" and "gui"
shared structures.

```
% Create the controls
controlLayout = uix.VBox( 'Parent', controlPanel, ...
   'Padding', 3, 'Spacing', 3 );
gui.ListBox = uicontrol( 'Style', 'list', ...
   'BackgroundColor', 'w', ...
   'Parent', controlLayout, ...
   'String', demoList(:), ...
   'Value', 1, ...
   'Callback', @onListSelection);
gui.HelpButton = uicontrol( 'Style', 'PushButton', ...
   'Parent', controlLayout, ...
   'String', 'Help for <demo>', ...
   'Callback', @onDemoHelp );
set( controlLayout, 'Heights', [-1 28] ); % Make the list fill the space
```

Finally, the view itself is simply an axes placed inside the view panel:

```
% Create the view
gui.ViewAxes = axes( 'Parent', gui.ViewPanel );
```

(Full source code for this application is available here:
[ view
| edit
| run ]
)

  

|  |  |  |  |  |
| --- | --- | --- | --- | --- |
|  | Application structure | [Top] | updateInterface |  |

© 2018 The MathWorks Ltd
• Terms of Use
• Patents
• Trademarks
