# Supplementary material for: Astrocytic Ca2+ signaling is reduced during sleep and is involved in the regulation of slow wave sleep
Source: Nat Commun. 2020 Jul 6;11:3240. doi: 10.1038/s41467-020-17062-2 (PMC7338360; doi:10.1038/s41467-020-17062-2)
Supplement: Supplementary file 8 — Supplementary Software [file 41467_2020_17062_MOESM8_ESM.zip › sleep_project_demo/gui_layout_toolbox/layoutdoc/User_guide2_1.html]

Position vs OuterPosition


|  |  |
| --- | --- |
| **User\_guide** |  |

  

## 2.1: Position vs OuterPosition

Typically one
would position some axes using their `OuterPosition` so that the
axis labels, title and other annotations are all contained within the
specified area. Sometimes, particularly if drawing images, one might want
to instead make the axes canvas (the white bit!) fill the specified area.
This is done by setting the `Position` property instead.

### For example:

```
figure
axes( 'Units', 'Normalized', 'OuterPosition', [0 0 1 1] )
```

```
figure
axes( 'Units', 'Normalized', 'Position', [0 0 1 1] )
```

  

|  |  |  |  |  |
| --- | --- | --- | --- | --- |
|  | Positioning axes | [Top] | Axes inside layouts |  |

© 2018 The MathWorks Ltd
• Terms of Use
• Patents
• Trademarks
