# Supplementary material for: Astrocytic Ca2+ signaling is reduced during sleep and is involved in the regulation of slow wave sleep
Source: Nat Commun. 2020 Jul 6;11:3240. doi: 10.1038/s41467-020-17062-2 (PMC7338360; doi:10.1038/s41467-020-17062-2)
Supplement: Supplementary file 8 — Supplementary Software [file 41467_2020_17062_MOESM8_ESM.zip › sleep_project_demo/gui_layout_toolbox/layoutdoc/index.html]

GUI Layout Toolbox


# GUI Layout Toolbox

**Version: 2.3.3**

GUI Layout Toolbox is a layout manager for creating MATLAB graphical user
interfaces that resize gracefully. The classes supplied can be used in
combination to produce virtually any user interface layout.

- Arrange MATLAB user interface components horizontally, vertically or in grids
- Mix fixed- and variable-size components
- Resize components interactively by dragging dividers
- Show and hide components using tabs and panels
- Show part of a large component in a scrollable panel

This toolbox was developed by
David Sampson and
Ben Tordoff from the
Consulting Services group
at MathWorks.

## Contents:

|  |  |  |
| --- | --- | --- |
|  | **Getting Started:** | Introductory notes and installation instructions |
|  | **Examples:** | A list of the examples that are provided in the documentation |
|  | **User guide:** | Describes how to use these tools |
|  | **Function reference:** | A list of the available functions |
|  | **Index:** | Alphabetical index of sections, functions and concepts |

  

© 2018 The MathWorks Ltd
• Terms of Use
• Patents
• Trademarks
