# Supplementary material for: Astrocytic Ca2+ signaling is reduced during sleep and is involved in the regulation of slow wave sleep
Source: Nat Commun. 2020 Jul 6;11:3240. doi: 10.1038/s41467-020-17062-2 (PMC7338360; doi:10.1038/s41467-020-17062-2)
Supplement: Supplementary file 8 — Supplementary Software [file 41467_2020_17062_MOESM8_ESM.zip › sleep_project_demo/gui_layout_toolbox/layoutdoc/User_guide7_3.html]

updateInterface


|  |  |
| --- | --- |
| **User\_guide** |  |

  

## 7.3: updateInterface

The second subfunction we will look at, `updateInterface`, uses the
current selections to update the interface. This uses the structure
"data" to update various parts of the interface. For this simple example this just means:

**1. Update the selected item in the listbox**

```
set( gui.ListBox, 'Value', data.SelectedDemo );
```

**2. Update the help button label**

```
demoName = data.DemoNames{ data.SelectedDemo };  
set( gui.HelpButton, 'String', ['Help for ',demoName] );
```

**3. Update the view panel title**

```
set( gui.ViewPanel, 'Title', sprintf( 'Viewing: %s', demoName ) );
```

**4. Update the ticked menu**

```
menus = get( gui.ViewMenu, 'Children' );
set( menus, 'Checked', 'off' );
% Use the name to work out which menu item should be ticked
whichMenu = strcmpi( demoName, get( menus, 'Label' ) );
set( menus(whichMenu), 'Checked', 'on' );
```


In general, this update function is called whenever the underlying
shared "data" structure is changed. This happens when the user clicks
a button, selects a list item or a menu. Next we will look at a typical
callback.

(Full source code for this application is available here:
[ view
| edit
| run ]
)

  

|  |  |  |  |  |
| --- | --- | --- | --- | --- |
|  | createInterface | [Top] | onListSelection |  |

© 2018 The MathWorks Ltd
• Terms of Use
• Patents
• Trademarks
