# Supplementary material for: Astrocytic Ca2+ signaling is reduced during sleep and is involved in the regulation of slow wave sleep
Source: Nat Commun. 2020 Jul 6;11:3240. doi: 10.1038/s41467-020-17062-2 (PMC7338360; doi:10.1038/s41467-020-17062-2)
Supplement: Supplementary file 8 — Supplementary Software [file 41467_2020_17062_MOESM8_ESM.zip › sleep_project_demo/gui_layout_toolbox/layoutdoc/Function_reference4_2.html]

uix.tracking


|  |  |
| --- | --- |
| **Function\_reference** |  |

  

## 4.2: uix.tracking

tracks anonymized usage data

`uix.tracking(` `p``,` `v``,` `id` `)`
:   tracks usage to the property p for the version v and identifier id using Google Analytics.

`uix.tracking(` `state` `)`
:   turns tracking on or off.

`state` `= uix.tracking(` `'query'` `)`
:   queries whether tracking is on or off.

### For example:

```
uix.tracking('UA-45678-9','1.2.3','featurename')
```

```
uix.tracking('on')
```

```
state = uix.tracking('query')
```

`state = 'on'`

  

|  |  |  |  |  |
| --- | --- | --- | --- | --- |
|  | uix.Empty | [Top] | layoutRoot |  |

© 2018 The MathWorks Ltd
• Terms of Use
• Patents
• Trademarks
