# Supplementary material for: Astrocytic Ca2+ signaling is reduced during sleep and is involved in the regulation of slow wave sleep
Source: Nat Commun. 2020 Jul 6;11:3240. doi: 10.1038/s41467-020-17062-2 (PMC7338360; doi:10.1038/s41467-020-17062-2)
Supplement: Supplementary file 8 — Supplementary Software [file 41467_2020_17062_MOESM8_ESM.zip › sleep_project_demo/gui_layout_toolbox/layoutdoc/User_guide1.html]

 


Understanding layouts


|  |  |
| --- | --- |
| **User\_guide** |  |

## 1: Understanding layouts

The purpose of a "layout" is to manage the positioning of one
or more user-interface components. This means that instead of
worrying about the exact position of each user interface component
you instead worry about the relative positioning of them; are they
arranged in a vertical list, horizontal list, a grid pattern, etc.

The best way to understand what layouts are and why they are a good user-interface
design tool is to see some examples. The sections below go through the
basics of building an interface using layouts and how to build complex
interfaces using them.

### Section contents:


- 1.1. Layout basics
- 1.2. Types of layout
- 1.3. Sizes and units
- 1.4. Layout hierarchies
- 1.5. Why use layouts?

  

|  |  |  |  |  |
| --- | --- | --- | --- | --- |
|  | User guide | [Top] | Layout basics |  |

© 2018 The MathWorks Ltd
• Terms of Use
• Patents
• Trademarks
