# Supplementary material for: Astrocytic Ca2+ signaling is reduced during sleep and is involved in the regulation of slow wave sleep
Source: Nat Commun. 2020 Jul 6;11:3240. doi: 10.1038/s41467-020-17062-2 (PMC7338360; doi:10.1038/s41467-020-17062-2)
Supplement: Supplementary file 8 — Supplementary Software [file 41467_2020_17062_MOESM8_ESM.zip › sleep_project_demo/gui_layout_toolbox/layoutdoc/Function_reference.html]

Function reference


|  |  |
| --- | --- |
| **GUI Layout Toolbox 2.3.3** |  |

# Function reference


|  |  |
| --- | --- |
| 1: Panels | |
| 1.1 uix.Panel | Arrange a single element inside a standard panel |
| 1.2 uix.CardPanel | Show one element (card) from a list |
| 1.3 uix.BoxPanel | Arrange a single element in a panel with boxed title |
| 1.4 uix.TabPanel | Arrange elements in a panel with tabs for selecting which is visible |
| 1.5 uix.ScrollingPanel | Arrange a single element inside a scrollable panel |
| 2: Boxes | |
| 2.1 uix.HBox | Arrange elements horizontally in a single row |
| 2.2 uix.VBox | Arrange elements vertically in a single column |
| 2.3 uix.HBoxFlex | Arrange elements horizontally with draggable dividers |
| 2.4 uix.VBoxFlex | Arrange elements vertically with draggable dividers |
| 2.5 uix.HButtonBox | Arrange buttons horizontally in a single row |
| 2.6 uix.VButtonBox | Arrange buttons vertically in a single column |
| 3: Grids | |
| 3.1 uix.Grid | Arrange elements in a two dimensional grid |
| 3.2 uix.GridFlex | Arrange elements in a two dimensional grid with draggable dividers |
| 4: Other functions | |
| 4.1 uix.Empty | Create an empty space |
| 4.2 uix.tracking | Track usage |
| 4.3 layoutRoot | Return the folder containing the GUI layout toolbox |

  
  

|  |  |  |  |  |
| --- | --- | --- | --- | --- |
|  | Front page | [Top] | uix.Panel |  |

© 2018 The MathWorks Ltd
• Terms of Use
• Patents
• Trademarks
