# Supplementary material for: Astrocytic Ca2+ signaling is reduced during sleep and is involved in the regulation of slow wave sleep
Source: Nat Commun. 2020 Jul 6;11:3240. doi: 10.1038/s41467-020-17062-2 (PMC7338360; doi:10.1038/s41467-020-17062-2)
Supplement: Supplementary file 8 — Supplementary Software [file 41467_2020_17062_MOESM8_ESM.zip › sleep_project_demo/gui_layout_toolbox/layoutdoc/User_guide1_2.html]

Types of layout


|  |  |
| --- | --- |
| **User\_guide** |  |

  

## 1.2: Types of layout

The layouts in this toolbox come in three forms:

1. **Panels**: show a single child element with some decoration. Other children
   of the layout are hidden from view. The visible child can be switched. Available
   panels include
   Panel,
   CardPanel,
   BoxPanel,
   TabPanel and
   ScrollingPanel.
2. **Boxes**: arrange children linearly in a single row or column. Available boxes include
   HBox,
   VBox,
   HBoxFlex and
   VBoxFlex.
3. **Grids**: (also known as tables) arrange children in a two-dimensional grid. Available grids include
   Grid and
   GridFlex.

  

|  |  |  |  |  |
| --- | --- | --- | --- | --- |
|  | Layout basics | [Top] | Sizes and units |  |

© 2018 The MathWorks Ltd
• Terms of Use
• Patents
• Trademarks
