# Supplementary material for: Astrocytic Ca2+ signaling is reduced during sleep and is involved in the regulation of slow wave sleep
Source: Nat Commun. 2020 Jul 6;11:3240. doi: 10.1038/s41467-020-17062-2 (PMC7338360; doi:10.1038/s41467-020-17062-2)
Supplement: Supplementary file 8 — Supplementary Software [file 41467_2020_17062_MOESM8_ESM.zip › sleep_project_demo/gui_layout_toolbox/layoutdoc/User_guide4_1.html]

Context help


|  |  |
| --- | --- |
| **User\_guide** |  |

  

## 4.1: Context help

When a `uix.BoxPanel` has its `HelpFcn`
filled in, a help button (?) is shown in the upper-right of the
title-bar. When the user clicks this button the specified function
is called.

### For example:

Here we hookup the HelpFcn to simply bring up
the MATLAB documentation for each command. Clicking any of the "?" buttons
will cause the MATLAB help browser to open.

```
f = figure( 'Name', 'uix.BoxPanel Help Example' );
b = uix.HBox( 'Parent', f );
uix.BoxPanel( 'Parent', b, 'Title', 'sin', 'HelpFcn', @(a,b) doc('sin') );
uix.BoxPanel( 'Parent', b, 'Title', 'cos', 'HelpFcn', @(a,b) doc('cos') );
uix.BoxPanel( 'Parent', b, 'Title', 'tan', 'HelpFcn', @(a,b) doc('tan') );
```

  

|  |  |  |  |  |
| --- | --- | --- | --- | --- |
|  | Advanced maneuvers with panels | [Top] | Minimize and maximize |  |

© 2018 The MathWorks Ltd
• Terms of Use
• Patents
• Trademarks
