# Supplementary material for: Astrocytic Ca2+ signaling is reduced during sleep and is involved in the regulation of slow wave sleep
Source: Nat Commun. 2020 Jul 6;11:3240. doi: 10.1038/s41467-020-17062-2 (PMC7338360; doi:10.1038/s41467-020-17062-2)
Supplement: Supplementary file 8 — Supplementary Software [file 41467_2020_17062_MOESM8_ESM.zip › sleep_project_demo/gui_layout_toolbox/layoutdoc/Function_reference2_3.html]

uix.HBoxFlex


|  |  |
| --- | --- |
| **Function\_reference** |  |

  

## 2.3: uix.HBoxFlex

  

Arrange elements horizontally with draggable dividers

`obj` `= uix.HBoxFlex(` `)`
:   creates a new horizontal box layout with draggable dividers and with all properties set to defaults.
    The output is a new layout object that can be used as the parent for other user-interface components.

`obj` `= uix.HBoxFlex(` `prop``,` `value``,` `...` `)`
:   also sets one or more property values.

### uix.HBoxFlex properties

| Property | Value | Description |
| --- | --- | --- |
| `BackgroundColor` | `colorspec` | Color to use for exposed areas of the layout background. This can be an RGB triple (e.g. [0 0 1]) or a colour name (e.g. 'b'). |
| `BeingDeleted` | on \| off | Deletion status. |
| `Contents` | empty GraphicsPlaceholder array \| array of graphics objects | Children within this layout, regardless of HandleVisibility. Note that this can only be set to permutations of itself. |
| `DeleteFcn` | `function_handle` | Function to call when the layout is being deleted. |
| `DividerMarkings` | on \| off | Show markings on the draggable dividers (default 'on'). |
| `MinimumWidths` | double vector | Minimum width in pixels of each of the children. The size of this vector must match the size of Contents. |
| `Padding` | positive integer | Number of pixels of extra space around the outside of the layout. |
| `Parent` | empty GraphicsPlaceholder array \| figure \| container | Parent of the layout. |
| `Position` | [x y w h] | Position (x,y) and size (w,h) within figure or container. |
| `Spacing` | positive integer | Number of pixels of extra space to leave between elements in the layout (default 5). |
| `Tag` | string | Tag to associate with layout. |
| `Type` | string | Type of graphics object. |
| `Units` | inches \| centimeters \| normalized \| points \| pixels \| characters | Position units. |
| `Visible` | on \| off | Visibility. |
| `Widths` | double vector | Width of each of the children. Positive entries indicate fixed sizes in pixels, negative values indicate relative weights for resizing. The size of this vector must always match the size of Contents. |

### For example:

```
f = figure( 'Name', 'uix.HBoxFlex example' );
b = uix.HBoxFlex( 'Parent', f );
uicontrol( 'Parent', b, 'Background', 'r' )
uicontrol( 'Parent', b, 'Background', 'b' )
uicontrol( 'Parent', b, 'Background', 'g' )
uicontrol( 'Parent', b, 'Background', 'y' )
set( b, 'Widths', [-1 100 -2 -1], 'Spacing', 5 );
```

  
**See also:**

- `uix.HBox` - for creating a horizontal arrangement
- `uix.VBoxFlex` - for creating a vertical arrangement with draggable dividers

  

|  |  |  |  |  |
| --- | --- | --- | --- | --- |
|  | uix.VBox | [Top] | uix.VBoxFlex |  |

© 2018 The MathWorks Ltd
• Terms of Use
• Patents
• Trademarks
