# Supplementary material for: Astrocytic Ca2+ signaling is reduced during sleep and is involved in the regulation of slow wave sleep
Source: Nat Commun. 2020 Jul 6;11:3240. doi: 10.1038/s41467-020-17062-2 (PMC7338360; doi:10.1038/s41467-020-17062-2)
Supplement: Supplementary file 8 — Supplementary Software [file 41467_2020_17062_MOESM8_ESM.zip › sleep_project_demo/gui_layout_toolbox/layoutdoc/Getting_Started.html]

Getting Started


|  |  |
| --- | --- |
| **GUI Layout Toolbox 2.3.3** |  |

# Getting Started

This chapter gives an overview of this package, including its purpose,
how to install it and any known issues at the time of release.


  


## Contents

- 1. What is GUI Layout Toolbox?
- 2. Compatibility considerations
- 3. Release notes
- 4. Installation
- 5. Support
- 6. Acknowledgements

  

|  |  |  |  |  |
| --- | --- | --- | --- | --- |
|  | Front page | [Top] | What is GUI Layout Toolbox? |  |

© 2018 The MathWorks Ltd
• Terms of Use
• Patents
• Trademarks
