# Supplementary material for: Astrocytic Ca2+ signaling is reduced during sleep and is involved in the regulation of slow wave sleep
Source: Nat Commun. 2020 Jul 6;11:3240. doi: 10.1038/s41467-020-17062-2 (PMC7338360; doi:10.1038/s41467-020-17062-2)
Supplement: Supplementary file 8 — Supplementary Software [file 41467_2020_17062_MOESM8_ESM.zip › sleep_project_demo/gui_layout_toolbox/layoutdoc/User_guide6.html]

 


Deploying GUIs using the MATLAB Compiler


|  |  |
| --- | --- |
| **User\_guide** |  |

## 6: Deploying GUIs using the MATLAB Compiler

Applications built using these layout tools can be deployed as standalone
executables using the MATLAB Compiler in
the same way as any other MATLAB application. There is, however, one thing to watch out for:

- You must explicitly include the "Resources" folder within the +uix package

#### Example

Below is a screen-shot of the `deploytool` setup
used to build the example application.
The "Resources" folder from the toolbox has been explicitly added as a
shared resource so that the mouse-pointers and panel icons continue to work.


  

|  |  |  |  |  |
| --- | --- | --- | --- | --- |
|  | Using layouts inside GUIDE GUIs | [Top] | A complete example |  |

© 2018 The MathWorks Ltd
• Terms of Use
• Patents
• Trademarks
