# Supplementary material for: Astrocytic Ca2+ signaling is reduced during sleep and is involved in the regulation of slow wave sleep
Source: Nat Commun. 2020 Jul 6;11:3240. doi: 10.1038/s41467-020-17062-2 (PMC7338360; doi:10.1038/s41467-020-17062-2)
Supplement: Supplementary file 8 — Supplementary Software [file 41467_2020_17062_MOESM8_ESM.zip › sleep_project_demo/gui_layout_toolbox/layoutdoc/helptoc.html]

Table of Contents


 **GUI Layout**  
  
  **Getting Started**  
     1. What is GUI Layout Toolbox?  
     2. Compatibility considerations  
     3. Release notes  
     4. Installation  
     5. Support  
     6. Acknowledgements  
  
  **Examples**  
  
  **User guide**  
     1. Understanding layouts  
     2. Positioning axes  
     3. Controlling visibility  
     4. Advanced maneuvers with panels  
     5. Using layouts inside GUIDE GUIs  
     6. Deploying GUIs using the MATLAB Compiler  
     7. A complete example  
  
  **Function reference**  
  
  **Index**
