# Supplementary material for: Astrocytic Ca2+ signaling is reduced during sleep and is involved in the regulation of slow wave sleep
Source: Nat Commun. 2020 Jul 6;11:3240. doi: 10.1038/s41467-020-17062-2 (PMC7338360; doi:10.1038/s41467-020-17062-2)
Supplement: Supplementary file 8 — Supplementary Software [file 41467_2020_17062_MOESM8_ESM.zip › sleep_project_demo/gui_layout_toolbox/layoutdoc/User_guide7.html]

 


A complete example


|  |  |
| --- | --- |
| **User\_guide** |  |

## 7: A complete example

The following example application uses many of the layout features
discussed above in order to create a good-looking user interface that
scales well when resized. It is not designed to showcase all the
layout functionality, but shows how callbacks are added to provide
user interaction. It also exemplifies separating the data from the GUI,
a fundamental part of creating modular and maintainable applications.

The full application is available here:

- View demoBrowser.m
- Edit demoBrowser.m
- Run demoBrowser.m

### Section contents:


- 7.1. Application structure
- 7.2. createInterface
- 7.3. updateInterface
- 7.4. onListSelection
- 7.5. Running it
- 7.6. Scalability

  

|  |  |  |  |  |
| --- | --- | --- | --- | --- |
|  | Deploying GUIs using the MATLAB Compiler | [Top] | Application structure |  |

© 2018 The MathWorks Ltd
• Terms of Use
• Patents
• Trademarks
