# Supplementary material for: Astrocytic Ca2+ signaling is reduced during sleep and is involved in the regulation of slow wave sleep
Source: Nat Commun. 2020 Jul 6;11:3240. doi: 10.1038/s41467-020-17062-2 (PMC7338360; doi:10.1038/s41467-020-17062-2)
Supplement: Supplementary file 8 — Supplementary Software [file 41467_2020_17062_MOESM8_ESM.zip › sleep_project_demo/gui_layout_toolbox/layoutdoc/Getting_Started1.html]

 


What is GUI Layout Toolbox?


|  |  |
| --- | --- |
| **Getting\_Started** |  |

## 1: What is GUI Layout Toolbox?

GUI Layout Toolbox provides a package of MATLAB objects that allow for complex
arrangement of graphical user interface elements within a figure window. The
main capabilities provided are:

- Automatic element arrangement horizontally, vertically or in grids
- Ability to specify fixed sizes or resizing weights for each element
- Ability to "nest" layouts to produce virtually any element arrangement
- Divider bars for user-resizing of elements

These element arrangement capabilities are designed to match those found as
standard in other user-interface toolkits such as Java Swing, GTK, QT etc.

  

|  |  |  |  |  |
| --- | --- | --- | --- | --- |
|  | Getting Started | [Top] | Compatibility considerations |  |

© 2018 The MathWorks Ltd
• Terms of Use
• Patents
• Trademarks
