# Supplementary material for: Astrocytic Ca2+ signaling is reduced during sleep and is involved in the regulation of slow wave sleep
Source: Nat Commun. 2020 Jul 6;11:3240. doi: 10.1038/s41467-020-17062-2 (PMC7338360; doi:10.1038/s41467-020-17062-2)
Supplement: Supplementary file 8 — Supplementary Software [file 41467_2020_17062_MOESM8_ESM.zip › sleep_project_demo/gui_layout_toolbox/layoutdoc/User_guide1_3.html]

Sizes and units


|  |  |
| --- | --- |
| **User\_guide** |  |

  

## 1.3: Sizes and units

In the previous section we noted that when using layouts
you never need to set the position or size of a user-interface
component yourself - the layouts take care of it. So how do
you control the sizes of the components?

Each layout that arranges multiple items within its drawing area
has a sizing property: for horizontal boxes this is `Widths`
and for vertical boxes `Heights`; for grids we have both `Widths`
and `Heights`. These all obey the same convention:

1. Positive numbers indicate sizes in pixels (similar to "pixel" units)
2. Negative numbers indicate a weighting for variable sizing (similar to "normalized" units)

By default all sizes are set to -1 (variable size with unit weighting).

Let's take a simple example:

```
f = figure();
layout = uix.HBox( 'Parent', f );
uicontrol( 'String', 'Button 1', 'Parent', layout );
uicontrol( 'String', 'Button 2', 'Parent', layout );
uicontrol( 'String', 'Button 3', 'Parent', layout );
```

We can set the middle element to be twice as wide as the others
(but still variable width) by setting its weight to -2 with the others at -1:

```
layout.Widths = [-1 -2 -1]
```

Alternatively we might want the first element to have a
fixed width of 100 pixels with the others filling any remaining
space equally:

```
layout.Widths = [100 -1 -1]
```

This ability to mix fixed and variable sized elements is
crucial in interface design. It really comes into its own when building
a hierarchy of layouts, described next.

  

---

### 1.3.1: Minimum sizes

Many of the multi-element layouts also provide a `MinimumWidths`
or `MinimumHeights`
property to prevent an element becoming too small. This is measured in
pixels and defaults to one pixel. Take care to ensure that the available
space is at least the sum of the minimum sizes, plus any padding and
spacing.

  

|  |  |  |  |  |
| --- | --- | --- | --- | --- |
|  | Types of layout | [Top] | Layout hierarchies |  |

© 2018 The MathWorks Ltd
• Terms of Use
• Patents
• Trademarks
