# Supplementary material for: Astrocytic Ca2+ signaling is reduced during sleep and is involved in the regulation of slow wave sleep
Source: Nat Commun. 2020 Jul 6;11:3240. doi: 10.1038/s41467-020-17062-2 (PMC7338360; doi:10.1038/s41467-020-17062-2)
Supplement: Supplementary file 8 — Supplementary Software [file 41467_2020_17062_MOESM8_ESM.zip › sleep_project_demo/gui_layout_toolbox/layoutdoc/User_guide7_5.html]

Running it


|  |  |
| --- | --- |
| **User\_guide** |  |

  

## 7.5: Running it

When the main function is launched (click
here)
it first creates the data, then the GUI, then updates the GUI using the data. At this point the
function exits and control is returned to the command prompt. Note, however,
that the GUI is still onscreen and will still respond to user interaction.

This works because the "shared" variables in the main function are not cleared when the
function exits. They are only cleared once the GUI is closed. This is a slightly
unusual, but very useful, feature of using nested functions for building
applications.

### For example:

```
>> demoBrowser
```

(Full source code for this application is available here:
[ view
| edit
| run ]
)

  

|  |  |  |  |  |
| --- | --- | --- | --- | --- |
|  | onListSelection | [Top] | Scalability |  |

© 2018 The MathWorks Ltd
• Terms of Use
• Patents
• Trademarks
