# Supplementary material for: Astrocytic Ca2+ signaling is reduced during sleep and is involved in the regulation of slow wave sleep
Source: Nat Commun. 2020 Jul 6;11:3240. doi: 10.1038/s41467-020-17062-2 (PMC7338360; doi:10.1038/s41467-020-17062-2)
Supplement: Supplementary file 8 — Supplementary Software [file 41467_2020_17062_MOESM8_ESM.zip › sleep_project_demo/gui_layout_toolbox/layoutdoc/User_guide4_3.html]

Dock and undock


|  |  |
| --- | --- |
| **User\_guide** |  |

  

## 4.3: Dock and undock

When a `uix.BoxPanel` has its `DockFcn`
filled in, a dock/undock button (↘/↗) is shown in the upper-right of the
title-bar. When the user clicks this button the specified function
is called. Since re-docking the panel into its previous parent
depends on the type of parent, it is up to the user to write
some code to actually extract or insert the panel.

The following simple example shows how to add dock/undock
functionality to a box full of panels. Save the code into
a file called "dockexample.m" to run it.

(The code for this example can be found here:
[ view
| edit
| run ]
)

#### Create the layout with three panels

Open a new figure window and add three panels.

```
function dockexample()
% Create the window and main layout
fig = figure( 'Name', 'Dockable GUI example', ...
              'NumberTitle', 'off', ...
              'Toolbar', 'none', ...
              'MenuBar', 'none', ...
              'CloseRequestFcn', @nCloseAll );
box = uix.HBox( 'Parent', fig );

% Add three panels to the box
panel{1} = uix.BoxPanel( 'Title', 'Panel 1', 'Parent', box );
panel{2} = uix.BoxPanel( 'Title', 'Panel 2', 'Parent', box );
panel{3} = uix.BoxPanel( 'Title', 'Panel 3', 'Parent', box );

% Add some contents  

uicontrol( 'Style', 'PushButton', 'String', 'Button 1', 'Parent', panel{1} );
uicontrol( 'Style', 'PushButton', 'String', 'Button 2', 'Parent', panel{2} );
uicontrol( 'Style', 'PushButton', 'String', 'Button 3', 'Parent', panel{3} );
```

#### Add the dock/undock callback

We set each panel to call the same dock/undock function.
This function is nested inside the main function so that it has access
to the main function's variables. A better way to do this is to make the
main function into a class, but this nested-function approach is fine
for simple applications.

Note that as soon as we set the "DockFcn" property the Dock/Undock
icon appears in the top-right of each panel. We use a cell-array to pass an
extra argument, the panel number, to the minimize function. This extra argument appears after the usual
`eventSource` and `eventData` arguments.

```
% Set the dock/undock callback  

set( panel{1}, 'DockFcn', {@nDock, 1} );
set( panel{2}, 'DockFcn', {@nDock, 2} );
set( panel{3}, 'DockFcn', {@nDock, 3} );

%-------------------------------------------------------------------------%  
 
    function nDock( eventSource, eventData, whichpanel )
        % Set the flag
        panel{whichpanel}.Docked = ~panel{whichpanel}.Docked;
        if panel{whichpanel}.Docked
            % Put it back into the layout
            newfig = get( panel{whichpanel}, 'Parent' );
            set( panel{whichpanel}, 'Parent', box );
            delete( newfig );
        else 
            % Take it out of the layout
            pos = getpixelposition( panel{whichpanel} );
            newfig = figure( ...
                'Name', get( panel{whichpanel}, 'Title' ), ...
                'NumberTitle', 'off', ...
                'MenuBar', 'none', ...
                'Toolbar', 'none', ...
                'CloseRequestFcn', {@nDock, whichpanel} );
            figpos = get( newfig, 'Position' );
            set( newfig, 'Position', [figpos(1,1:2), pos(1,3:4)] );
            set( panel{whichpanel}, 'Parent', newfig, ...
                'Units', 'Normalized', ...
                'Position', [0 0 1 1] );
        end 
    end % nDock
```

#### Add the close callback

If the user closes the main window we need to also close any
other windows that were created. This can be done by finding
the window that contains each panel and deleting it.

```
%-------------------------------------------------------------------------%  
 
   function nCloseAll( ~, ~ )
      for ii=1:numel( panel )
         if isvalid( panel{ii} ) && ~strcmpi( panel{ii}.BeingDeleted, 'on' )
            figh = ancestor( panel{ii}, 'figure' );
            delete( figh );
         end 
      end 
        
   end % nCloseAll
end % Main function
```

#### Click the dock buttons

Undocking the middle panel causes the other two to fill the
vacated space. The undocked panel appears in its own window, with the
"Undock" icon replaced by a "Dock" icon.

.

Re-docking the panel would
cause it to be appended to the right of the list in the original window. Closing the main window
causes all panels, docked or undocked, and their enclosing windows to be closed.

  

|  |  |  |  |  |
| --- | --- | --- | --- | --- |
|  | Minimize and maximize | [Top] | Using layouts inside GUIDE GUIs |  |

© 2018 The MathWorks Ltd
• Terms of Use
• Patents
• Trademarks
