# Supplementary material for: Astrocytic Ca2+ signaling is reduced during sleep and is involved in the regulation of slow wave sleep
Source: Nat Commun. 2020 Jul 6;11:3240. doi: 10.1038/s41467-020-17062-2 (PMC7338360; doi:10.1038/s41467-020-17062-2)
Supplement: Supplementary file 8 — Supplementary Software [file 41467_2020_17062_MOESM8_ESM.zip › sleep_project_demo/gui_layout_toolbox/layoutdoc/User_guide7_6.html]

Scalability


|  |  |
| --- | --- |
| **User\_guide** |  |

  

## 7.6: Scalability

As applications get bigger the code gets more complex. The simple
application structure used here does not scale well to large applications,
however some small adjustments can make life much better:

- Convert the "data" structure into a handle object. This allows
  a single "data" object to be shared between mutliple graphical interfaces, and in turn means that the interface
  need not be built as a single monolithic entity.
- Use the events system to trigger updates
  to specific parts of the GUI in response to bits of the data object changing. This removes the need
  for a single large "UpdateInterface" function and reduces coupling between parts of the interface. For example,
  the "SelectedDemo" property would have an associated event such that when it is changed by a callback (or from the command-line) it
  notifies other interface components of the change. Each interface component (or group thereof) can
  just listen for the events that affect it.

Advice on how to build large-scale applications is beyond the scope
of this document. If you need help in this area, please contact your MathWorks
account manager who will be able to put you in touch with a technical specialist.

  

|  |  |  |  |  |
| --- | --- | --- | --- | --- |
|  | Running it | [Top] | Front page |  |

© 2018 The MathWorks Ltd
• Terms of Use
• Patents
• Trademarks
>
