# Supplementary material for: Astrocytic Ca2+ signaling is reduced during sleep and is involved in the regulation of slow wave sleep
Source: Nat Commun. 2020 Jul 6;11:3240. doi: 10.1038/s41467-020-17062-2 (PMC7338360; doi:10.1038/s41467-020-17062-2)
Supplement: Supplementary file 8 — Supplementary Software [file 41467_2020_17062_MOESM8_ESM.zip › sleep_project_demo/gui_layout_toolbox/layoutdoc/User_guide.html]

User guide


|  |  |
| --- | --- |
| **GUI Layout Toolbox 2.3.3** |  |

# User guide

This chapter describes the purpose and overall design of the layout
tools that the toolbox provides. It also provides some examples of
creating some user interfaces using layouts.


  


## Contents

- 1. Understanding layouts
- 2. Positioning axes
- 3. Controlling visibility
- 4. Advanced maneuvers with panels
- 5. Using layouts inside GUIDE GUIs
- 6. Deploying GUIs using the MATLAB Compiler
- 7. A complete example

  

|  |  |  |  |  |
| --- | --- | --- | --- | --- |
|  | Front page | [Top] | Understanding layouts |  |

© 2018 The MathWorks Ltd
• Terms of Use
• Patents
• Trademarks
