# Supplementary material for: Astrocytic Ca2+ signaling is reduced during sleep and is involved in the regulation of slow wave sleep
Source: Nat Commun. 2020 Jul 6;11:3240. doi: 10.1038/s41467-020-17062-2 (PMC7338360; doi:10.1038/s41467-020-17062-2)
Supplement: Supplementary file 8 — Supplementary Software [file 41467_2020_17062_MOESM8_ESM.zip › sleep_project_demo/gui_layout_toolbox/layoutdoc/User_guide3_1.html]

Visible example


|  |  |
| --- | --- |
| **User\_guide** |  |

  

## 3.1: Visible example

(The code for this example can be found here:
[ view
| edit
| run ]
)

This example shows how the `Visible` property can be
used to hide whole sections of an interface.

Open a window and add a panel

```
fig = figure( 'Name', 'Visible example', ...
    'Position', [100 100 150 250], ...
    'MenuBar', 'none', ...
    'ToolBar', 'none', ...
    'NumberTitle', 'off' );
panel = uix.BoxPanel( 'Parent', fig, 'Title', 'Panel' );
```


Put some buttons inside the panel

```
box = uix.VButtonBox( 'Parent', panel );
uicontrol( 'Parent', box, 'String', 'Button 1' );
uicontrol( 'Parent', box, 'String', 'Button 2' );
uicontrol( 'Parent', box, 'String', 'Button 3', 'Visible', 'off' );
uicontrol( 'Parent', box, 'String', 'Button 4' );
uicontrol( 'Parent', box, 'String', 'Button 5', 'Visible', 'off' );
uicontrol( 'Parent', box, 'String', 'Button 6' );
```


Try hiding the panel

```
set( panel, 'Visible', 'off' );
```


Try showing the panel. Note that the original `Visible` state of each button is remembered.

```
set( panel, 'Visible', 'on' );
```

  

|  |  |  |  |  |
| --- | --- | --- | --- | --- |
|  | Controlling visibility | [Top] | Advanced maneuvers with panels |  |

© 2018 The MathWorks Ltd
• Terms of Use
• Patents
• Trademarks
