# Supplementary material for: Astrocytic Ca2+ signaling is reduced during sleep and is involved in the regulation of slow wave sleep
Source: Nat Commun. 2020 Jul 6;11:3240. doi: 10.1038/s41467-020-17062-2 (PMC7338360; doi:10.1038/s41467-020-17062-2)
Supplement: Supplementary file 8 — Supplementary Software [file 41467_2020_17062_MOESM8_ESM.zip › sleep_project_demo/gui_layout_toolbox/layoutdoc/User_guide4.html]

 


Advanced maneuvers with panels


|  |  |
| --- | --- |
| **User\_guide** |  |

## 4: Advanced maneuvers with panels

The `uix.BoxPanel` provides some extra buttons
and callbacks that can be used to design advanced user-interface
layouts. In particular, the sections below illustrate how to create
interfaces with context help, and panels that can be minimized or undocked.

### Section contents:


- 4.1. Context help
- 4.2. Minimize and maximize
- 4.3. Dock and undock

  

|  |  |  |  |  |
| --- | --- | --- | --- | --- |
|  | Visible example | [Top] | Context help |  |

© 2018 The MathWorks Ltd
• Terms of Use
• Patents
• Trademarks
