# Supplementary material for: Astrocytic Ca2+ signaling is reduced during sleep and is involved in the regulation of slow wave sleep
Source: Nat Commun. 2020 Jul 6;11:3240. doi: 10.1038/s41467-020-17062-2 (PMC7338360; doi:10.1038/s41467-020-17062-2)
Supplement: Supplementary file 8 — Supplementary Software [file 41467_2020_17062_MOESM8_ESM.zip › sleep_project_demo/gui_layout_toolbox/layoutdoc/Function_reference1_4.html]

uix.TabPanel


|  |  |
| --- | --- |
| **Function\_reference** |  |

  

## 1.4: uix.TabPanel

  

Arrange elements in a panel with tabs for selecting which is visible

`obj` `= uix.TabPanel(` `)`
:   creates a panel with tabs along one edge
    to allow selection between the different child objects contained.

`obj` `= uix.TabPanel(` `prop``,` `value``,` `...` `)`
:   also sets one or more property values.

### uix.TabPanel properties

| Property | Value | Description |
| --- | --- | --- |
| `BackgroundColor` | `colorspec` | Color to use for exposed areas of the layout background. This can be an RGB triple (e.g. [0 0 1]) or a colour name (e.g. 'b'). |
| `BeingDeleted` | on \| off | Deletion status. |
| `Contents` | empty GraphicsPlaceholder array \| array of graphics objects | Children within this layout, regardless of HandleVisibility. Note that this can only be set to permutations of itself. |
| `DeleteFcn` | `function_handle` | Function to call when the layout is being deleted. |
| `FontAngle` | normal \| italic \| oblique | Title font angle. |
| `FontName` | string | Title font name (e.g. Arial, Helvetica etc). |
| `FontSize` | positive integer | Title font size. |
| `FontUnits` | inches \| centimeters \| normalized \| points \| pixels | Title font units for measuring size. |
| `FontWeight` | light \| normal \| demi \| bold | Title font weight. |
| `ForegroundColor` | `colorspec` | Title font color and/or color of 2-D border line. |
| `HighlightColor` | `colorspec` | 3-D frame highlight color. |
| `ShadowColor` | `colorspec` | 3-D frame shadow color. |
| `Padding` | positive integer | Number of pixels of extra space around the outside of the layout. |
| `Parent` | empty GraphicsPlaceholder array \| figure \| container | Parent of the layout. |
| `Position` | [x y w h] | Position (x,y) and size (w,h) within figure or container. |
| `Selection` | positive integer or empty | Which child is visible. |
| `SelectionChangedFcn` | `function_handle` | Function to call when the selected tab is changed. The event-data supplied has fields `OldValue` and `NewValue` giving the previously selected and newly selected tab indices. |
| `TabContextMenus` | cell array of context menus | The context menu (or []) for each tab. |
| `TabEnables` | cell array of on \| off | A list of the enabled state of each tab (default is all 'on'). |
| `TabTitles` | cell array of strings | A list of the names of the tabs with one entry per tab. |
| `TabWidth` | positive integer | Width of each tab in pixels (default 50). |
| `Tag` | string | Tag to associate with layout. |
| `Type` | string | Type of graphics object. |
| `Units` | inches \| centimeters \| normalized \| points \| pixels \| characters | Position units. |
| `Visible` | on \| off | Visibility. |

### For example:

```
f = figure();
p = uix.TabPanel( 'Parent', f, 'Padding', 5 );
uicontrol( 'Parent', p, 'Background', 'r' );
uicontrol( 'Parent', p, 'Background', 'b' );
uicontrol( 'Parent', p, 'Background', 'g' );
p.TabTitles = {'Red', 'Blue', 'Green'};
p.Selection = 2;
```

  
**See also:**

- `uix.Panel` - for creating a more standard panel
- `uix.CardPanel` - for creating an undecorated panel
- `uix.BoxPanel` - for creating a panel with boxed title

  

|  |  |  |  |  |
| --- | --- | --- | --- | --- |
|  | uix.BoxPanel | [Top] | uix.ScrollingPanel |  |

© 2018 The MathWorks Ltd
• Terms of Use
• Patents
• Trademarks
