# Supplementary material for: Astrocytic Ca2+ signaling is reduced during sleep and is involved in the regulation of slow wave sleep
Source: Nat Commun. 2020 Jul 6;11:3240. doi: 10.1038/s41467-020-17062-2 (PMC7338360; doi:10.1038/s41467-020-17062-2)
Supplement: Supplementary file 8 — Supplementary Software [file 41467_2020_17062_MOESM8_ESM.zip › sleep_project_demo/gui_layout_toolbox/layoutdoc/Getting_Started3.html]

 


Release notes


|  |  |
| --- | --- |
| **Getting\_Started** |  |

## 3: Release notes

The GUI Layout Toolbox version numbers take the form `major.minor.iter`. The
current version you have installed can be checked by typing `ver`
at the MATLAB command prompt.

### Section contents:

- 3.1. Version 2.3.3
- 3.2. Version 2.3.2
- 3.3. Version 2.3.1
- 3.4. Version 2.3
- 3.5. Version 2.2.2
- 3.6. Version 2.2.1
- 3.7. Version 2.2
- 3.8. Version 2.1.2
- 3.9. Version 2.1.1
- 3.10. Version 2.1


  

---

## 3.1: Version 2.3.3

- Released 25 October 2018
- Updated for compatibility with R2018b axes toolbars
- Fixed G1804440 "Flex containers position axes incorrectly when ActivePositionProperty is outerposition"
- Updated documentation

  

---

## 3.2: Version 2.3.2

- Released 1 May 2018
- Set tooltip strings for BoxPanel dock/undock, minimize/maximize, help and close buttons
- Set default padding for flex containers to 5 so that dividers are visible
- Scroll uix.ScrollingPanel using mouse wheel for variable-sized contents
- Fixed G1358897 "BoxPanel title is truncated on Mac high DPI display"
- Fixed G1695618 "Warning on mouse motion while deleting HBoxFlex, VBoxFlex or GridFlex"
- Updated documentation

  

---

## 3.3: Version 2.3.1

- Released 31 January 2017
- Specify minimum width and height of contents in uix.ScrollingPanel
- Update contents position while dragging uix.ScrollingPanel scrollbox
- Scroll uix.ScrollingPanel using mouse wheel
- Updated toolbox logo
- Updated documentation

  

---

## 3.4: Version 2.3

- Released 24 November 2016
- Added scrolling panel
- Expand and collapse box panel by clicking on title
- Fixed G1493103 "Error on construction behavior is inconstistent with builtin objects"
- Updated documentation

  

---

## 3.5: Version 2.2.2

- Released 22 August 2016
- Fixed G1175938 "Cannot use data cursor mode with GUI Layout Toolbox containers"
- Fixed G1367337 "Update flex container pointer on mouse press event"
- Fixed G1380756 "Space behind TabPanel tabs should match parent color"
- Updated documentation
- Added anonymous tracking of version, operating system and usage to help
  us prioritize the improvements we should work on

  

---

## 3.6: Version 2.2.1

- Released 26 February 2016
- Fixed G1346921 "Mouse pointer gets confused when moving between adjacent flex containers"
- Fixed G1357340 "BoxPanel property ForegroundColor is initialized incorrectly"

  

---

## 3.7: Version 2.2

- Released 18 December 2015
- Improved box panel title bar appearance
- Changed selection behavior of uix.TabGroup to match that of uitabgroup when the selected tab is removed
- Fixed G1253937 "uix.TabPanel/redrawTabs fails" (R2015b)
- Fixed G1292238 "uix.BoxPanel/redrawBorders fails" (R2015b)
- Fixed G1330841 "mouse-over-divider detection does not work for docked figures" (all)
- Fixed G1332109 "uix.Empty background color does not match that of its Parent" (all)
- Fixed G1334867 "cannot add axes to container" (R2016a prerelease)
- Removed internal helper classes uix.AncestryObserver, uix.LocationObserver, uix.VisibilityObserver
- Updated documentation

  

---

## 3.8: Version 2.1.2

- Released 29 May 2015
- Fixed G1250248 "uix.Empty becomes visible in a panel"
- Fixed G1250249 "missing property Selection of uix.BoxPanel"
- Fixed G1250808 "uix.TabPanel context menus are orphaned when reparenting to a different figure"
- Updated documentation

  

---

## 3.9: Version 2.1.1

- Released 15 May 2015
- Added context menus on uix.TabPanel tab labels (G1245669)
- Fixed G1164656 "cannot set relative tab widths"
- Fixed G1019441 "property RowSizes of uiextras.GridFlex sets heights not widths"
- Fixed G1165274 "missing properties RowSizes, MinimumRowSizes, ColumnSizes,
  MinimumColumnSizes of uiextras.Grid, uiextras.GridFlex"
- Fixed G1218142 "contents are lost when reordering via property Children"
- Protected against G1136196 "segv when setting child visibility from 'off' to 'on' in response to being reparented"
- Updated documentation

  

---

## 3.10: Version 2.1

- Released 2 October 2014
- Initial version for MATLAB R2014b
- Versions 2.0.x were for prerelease testing

  

|  |  |  |  |  |
| --- | --- | --- | --- | --- |
|  | Compatibility considerations | [Top] | Installation |  |

© 2018 The MathWorks Ltd
• Terms of Use
• Patents
• Trademarks
