# Supplementary material for: Astrocytic Ca2+ signaling is reduced during sleep and is involved in the regulation of slow wave sleep
Source: Nat Commun. 2020 Jul 6;11:3240. doi: 10.1038/s41467-020-17062-2 (PMC7338360; doi:10.1038/s41467-020-17062-2)
Supplement: Supplementary file 8 — Supplementary Software [file 41467_2020_17062_MOESM8_ESM.zip › sleep_project_demo/gui_layout_toolbox/layoutdoc/Function_reference1_5.html]

uix.ScrollingPanel


|  |  |
| --- | --- |
| **Function\_reference** |  |

  

## 1.5: uix.ScrollingPanel

  

Arrange a single element inside a panel and provide scrollbars if the panel is smaller than the element

`obj` `= uix.ScrollingPanel(` `)`
:   creates a new scrolling panel.

`obj` `= uix.ScrollingPanel(` `prop``,` `value``,` `...` `)`
:   also sets one or more property values.

### uix.ScrollingPanel properties

| Property | Value | Description |
| --- | --- | --- |
| `BackgroundColor` | `colorspec` | Color to use for exposed areas of the layout background. This can be an RGB triple (e.g. [0 0 1]) or a colour name (e.g. 'b'). |
| `BeingDeleted` | on \| off | Deletion status. |
| `Contents` | empty GraphicsPlaceholder array \| array of graphics objects | Children within this layout, regardless of HandleVisibility. Note that this can only be set to permutations of itself. |
| `DeleteFcn` | `function_handle` | Function to call when the layout is being deleted. |
| `Heights` | double vector | Height of each of the children. Positive entries indicate fixed sizes in pixels, negative values indicate relative weights for resizing. The size of this vector must always match the size of Contents. |
| `HorizontalOffsets` | double vector | Horizontal offset of each of the children. Each value is limited to between 0 and the difference between the width of child and the width of the panel. The size of this vector must always match the size of Contents. |
| `HorizontalSteps` | positive double vector | Horizontal slider step for each of the children. The size of this vector must always match the size of Contents. |
| `MinimumHeights` | double vector | Minimum height in pixels of each of the children. The size of this vector must match the size of Contents. |
| `MinimumWidths` | double vector | Minimum width in pixels of each of the children. The size of this vector must match the size of Contents. |
| `MouseWheelEnabled` | on \| off | Mouse wheel scrolling status (default 'on'). |
| `Padding` | positive integer | This property is not honored by this class. |
| `Parent` | empty GraphicsPlaceholder array \| figure \| container | Parent of the layout. |
| `Position` | [x y w h] | Position (x,y) and size (w,h) within figure or container. |
| `Selection` | positive integer or empty | Which child is visible. |
| `Tag` | string | Tag to associate with layout. |
| `Type` | string | Type of graphics object. |
| `Units` | inches \| centimeters \| normalized \| points \| pixels \| characters | Position units. |
| `VerticalOffsets` | double vector | Vertical offset of each of the children. Each value is limited to between 0 and the difference between the height of child and the height of the panel. The size of this vector must always match the size of Contents. |
| `VerticalSteps` | positive double vector | Vertical slider step for each of the children. The size of this vector must always match the size of Contents. |
| `Visible` | on \| off | Visibility. |
| `Widths` | double vector | Width of each of the children. Positive entries indicate fixed sizes in pixels, negative values indicate relative weights for resizing. The size of this vector must always match the size of Contents. |

### For example:

```
f = figure( 'Name', 'uix.ScrollingPanel Help Example' );
f.Position(3:4) = 400;
p = uix.ScrollingPanel( 'Parent', f );
a = axes( 'Parent', p );
[x, y, z] = peaks();
surf( a, x, y, z )
a.ActivePositionProperty = 'position';
set( p, 'Widths', 600, 'Heights', 600, 'HorizontalOffsets', 100, 'VerticalOffsets', 100 )
```

  
**See also:**

- `uix.Panel` - for creating a standard panel
- `uix.CardPanel` - for creating an undecorated panel
- `uix.BoxPanel` - for creating a more boxy panel

  

|  |  |  |  |  |
| --- | --- | --- | --- | --- |
|  | uix.TabPanel | [Top] | uix.HBox |  |

© 2018 The MathWorks Ltd
• Terms of Use
• Patents
• Trademarks
