# Supplementary material for: Astrocytic Ca2+ signaling is reduced during sleep and is involved in the regulation of slow wave sleep
Source: Nat Commun. 2020 Jul 6;11:3240. doi: 10.1038/s41467-020-17062-2 (PMC7338360; doi:10.1038/s41467-020-17062-2)
Supplement: Supplementary file 8 — Supplementary Software [file 41467_2020_17062_MOESM8_ESM.zip › sleep_project_demo/gui_layout_toolbox/layoutdoc/User_guide1_1.html]

Layout basics


|  |  |
| --- | --- |
| **User\_guide** |  |

  

## 1.1: Layout basics

To see how layouts work, let's use the most basic layout,
a horizontal list (or box). We first create a window:

```
f = figure();
```

Now let's create the horizontal layout and add it to the figure. Note
that in common with other MATLAB graphics objects, one object is added to
another by setting the `Parent` property - this will automatically
adjust the list of `Children` in the parent object. The job of a
horizontal box layout is to arrange its contents in a horizontal line,
setting the position of each element to best fill the space:

```
layout = uix.HBox( 'Parent', f );
```

Nothing's changed! That's because the layout is for arranging
other user-interface components - it doesn't draw anything itself. Let's
add some buttons. Note how after creating each button the existing contents
of the box make room for the new addition; we don't need to set the position of
any user-interface component!

```
uicontrol( 'String', 'Button 1', 'Parent', layout );
uicontrol( 'String', 'Button 2', 'Parent', layout );
uicontrol( 'String', 'Button 3', 'Parent', layout );
```

..

Other layouts work in exactly the same way, although visually
the end-result is quite different:

```
f = figure();
layout = uix.VBox( 'Parent', f );
uicontrol( 'String', 'Button 1', 'Parent', layout );
uicontrol( 'String', 'Button 2', 'Parent', layout );
uicontrol( 'String', 'Button 3', 'Parent', layout );
```


```
f = figure();
layout = uix.TabPanel( 'Parent', f );
uicontrol( 'String', 'Button 1', 'Parent', layout );
uicontrol( 'String', 'Button 2', 'Parent', layout );
uicontrol( 'String', 'Button 3', 'Parent', layout );
```

  

|  |  |  |  |  |
| --- | --- | --- | --- | --- |
|  | Understanding layouts | [Top] | Types of layout |  |

© 2018 The MathWorks Ltd
• Terms of Use
• Patents
• Trademarks
