# Supplementary material for: Astrocytic Ca2+ signaling is reduced during sleep and is involved in the regulation of slow wave sleep
Source: Nat Commun. 2020 Jul 6;11:3240. doi: 10.1038/s41467-020-17062-2 (PMC7338360; doi:10.1038/s41467-020-17062-2)
Supplement: Supplementary file 8 — Supplementary Software [file 41467_2020_17062_MOESM8_ESM.zip › sleep_project_demo/gui_layout_toolbox/layoutdoc/Examples.html]

Examples


|  |  |
| --- | --- |
| **GUI Layout Toolbox 2.3.3** |  |

# Examples

The following examples are provided as part of this documentation. Click
the name of any example to go to the corresponding page.

#### Basics

Layout hierarchy
:   Using layouts inside layouts to produce more complex designs

#### Using axes

Positioning axes
:   Using the different axes position properties.

Axes legends and colorbars
:   How to work with axes that may also have legends and/or colorbars.

#### Using panels

Context help
:   Adding context-sensitive help using panels.

Minimize and maximize
:   Creating a user interface with panels that can be minimized and
    maximized.

Dock and undock
:   Creating a user interface with panels that can be undocked into
    separate windows.

#### Visible

Show and hide
:   Showing and hiding a layout and its contents.

#### Applications

Building a complete application
:   Using layouts together to produce a complete application.

  
  

|  |  |  |  |  |
| --- | --- | --- | --- | --- |
|  | Front page | [Top] | Front page |  |

© 2018 The MathWorks Ltd
• Terms of Use
• Patents
• Trademarks
