# Supplementary material for: Astrocytic Ca2+ signaling is reduced during sleep and is involved in the regulation of slow wave sleep
Source: Nat Commun. 2020 Jul 6;11:3240. doi: 10.1038/s41467-020-17062-2 (PMC7338360; doi:10.1038/s41467-020-17062-2)
Supplement: Supplementary file 8 — Supplementary Software [file 41467_2020_17062_MOESM8_ESM.zip › sleep_project_demo/gui_layout_toolbox/layoutdoc/Function_reference4_1.html]

uix.Empty


|  |  |
| --- | --- |
| **Function\_reference** |  |

  

## 4.1: uix.Empty

Create an empty space

`obj` `= uix.Empty(` `)`
:   creates an empty space object that can be
    used in layouts to add gaps between other elements.

`obj` `= uix.Empty(` `param``,` `value``,` `...` `)`
:   also sets one or more property values.

### uix.Empty properties

The empty space is achieved using a container that monitors
its parent's color and changes its own to match.

| Property | Value | Description |
| --- | --- | --- |
| `BeingDeleted` | on \| off | Deletion status. |
| `HandleVisibility` | on \| off | Visibility of the object. |
| `Parent` | empty GraphicsPlaceholder array \| figure \| container | Parent of the object. |
| `Position` | [x y w h] | Position (x,y) and size (w,h) within figure or container. |
| `Tag` | string | Tag to associate with object. |
| `Type` | string | Type of graphics object. |
| `Units` | inches \| centimeters \| normalized \| points \| pixels \| characters | Position units. |
| `Visible` | on \| off | Visibility. |

### For example:

```
f = figure();
box = uix.HBox( 'Parent', f, 'Spacing', 5 );
uicontrol( 'Parent', box, 'Background', 'r' )
uix.Empty( 'Parent', box )
uicontrol( 'Parent', box, 'Background', 'g' )
```

  
**See also:**

- `uix.HBox` - for arranging widgets horizontally

  

|  |  |  |  |  |
| --- | --- | --- | --- | --- |
|  | uix.GridFlex | [Top] | uix.tracking |  |

© 2018 The MathWorks Ltd
• Terms of Use
• Patents
• Trademarks
