# Supplementary material for: Astrocytic Ca2+ signaling is reduced during sleep and is involved in the regulation of slow wave sleep
Source: Nat Commun. 2020 Jul 6;11:3240. doi: 10.1038/s41467-020-17062-2 (PMC7338360; doi:10.1038/s41467-020-17062-2)
Supplement: Supplementary file 8 — Supplementary Software [file 41467_2020_17062_MOESM8_ESM.zip › sleep_project_demo/gui_layout_toolbox/layoutdoc/User_guide2.html]

 


Positioning axes


|  |  |
| --- | --- |
| **User\_guide** |  |

## 2: Positioning axes

Unlike other MATLAB user interface components, `axes`
have two position properties: `Position` and
`OuterPosition`. This means one has some extra
options as to how the layout will arrange the axes.

### Section contents:


- 2.1. Position vs OuterPosition
- 2.2. Axes inside layouts
- 2.3. Colorbars and legends

  

|  |  |  |  |  |
| --- | --- | --- | --- | --- |
|  | Why use layouts? | [Top] | Position vs OuterPosition |  |

© 2018 The MathWorks Ltd
• Terms of Use
• Patents
• Trademarks
