# Supplementary material for: Astrocytic Ca2+ signaling is reduced during sleep and is involved in the regulation of slow wave sleep
Source: Nat Commun. 2020 Jul 6;11:3240. doi: 10.1038/s41467-020-17062-2 (PMC7338360; doi:10.1038/s41467-020-17062-2)
Supplement: Supplementary file 8 — Supplementary Software [file 41467_2020_17062_MOESM8_ESM.zip › sleep_project_demo/gui_layout_toolbox/layoutdoc/Function_reference1_2.html]

uix.CardPanel


|  |  |
| --- | --- |
| **Function\_reference** |  |

  

## 1.2: uix.CardPanel

  

Show one element (card) from a list

`obj` `= uix.CardPanel(` `)`
:   creates a new card panel which allows
    selection between the different child objects contained, making the
    selected child fill the space available and all other children
    invisible. This is commonly used for creating wizards or quick
    switching between different views of a single data-set.

`obj` `= uix.CardPanel(` `prop``,` `value``,` `...` `)`
:   also sets one or more property values.

### uix.CardPanel properties

| Property | Value | Description |
| --- | --- | --- |
| `BackgroundColor` | `colorspec` | Color to use for exposed areas of the layout background. This can be an RGB triple (e.g. [0 0 1]) or a colour name (e.g. 'b'). |
| `BeingDeleted` | on \| off | Deletion status. |
| `Contents` | empty GraphicsPlaceholder array \| array of graphics objects | Children within this layout, regardless of HandleVisibility. Note that this can only be set to permutations of itself. |
| `DeleteFcn` | `function_handle` | Function to call when the layout is being deleted. |
| `Padding` | positive integer | Number of pixels of extra space around the outside of the layout. |
| `Parent` | empty GraphicsPlaceholder array \| figure \| container | Parent of the layout. |
| `Position` | [x y w h] | Position (x,y) and size (w,h) within figure or container. |
| `Selection` | positive integer or empty | Which child is visible. |
| `Tag` | string | Tag to associate with layout. |
| `Type` | string | Type of graphics object. |
| `Units` | inches \| centimeters \| normalized \| points \| pixels \| characters | Position units. |
| `Visible` | on \| off | Visibility. |

### For example:

```
f = figure();
p = uix.CardPanel( 'Parent', f, 'Padding', 5 );
uicontrol( 'Parent', p, 'Background', 'r' );
uicontrol( 'Parent', p, 'Background', 'b' );
uicontrol( 'Parent', p, 'Background', 'g' );
p.Selection = 2;
```

  
**See also:**

- `uix.Panel` - for creating a standard panel
- `uix.BoxPanel` - for creating a more boxy panel
- `uix.TabPanel` - for a panel with tabs for selecting the visible child

  

|  |  |  |  |  |
| --- | --- | --- | --- | --- |
|  | uix.Panel | [Top] | uix.BoxPanel |  |

© 2018 The MathWorks Ltd
• Terms of Use
• Patents
• Trademarks
