# Supplementary material for: Astrocytic Ca2+ signaling is reduced during sleep and is involved in the regulation of slow wave sleep
Source: Nat Commun. 2020 Jul 6;11:3240. doi: 10.1038/s41467-020-17062-2 (PMC7338360; doi:10.1038/s41467-020-17062-2)
Supplement: Supplementary file 8 — Supplementary Software [file 41467_2020_17062_MOESM8_ESM.zip › sleep_project_demo/gui_layout_toolbox/layoutdoc/Getting_Started4.html]

 


Installation


|  |  |
| --- | --- |
| **Getting\_Started** |  |

## 4: Installation

GUI Layout Toolbox is provided as a MATLAB toolbox file (`.mltbx`).

For instructions on installing and uninstalling, see the section on
managing toolboxes
in the MATLAB documentation.

  

|  |  |  |  |  |
| --- | --- | --- | --- | --- |
|  | Release notes | [Top] | Support |  |

© 2018 The MathWorks Ltd
• Terms of Use
• Patents
• Trademarks
