# Supplementary material for: Astrocytic Ca2+ signaling is reduced during sleep and is involved in the regulation of slow wave sleep
Source: Nat Commun. 2020 Jul 6;11:3240. doi: 10.1038/s41467-020-17062-2 (PMC7338360; doi:10.1038/s41467-020-17062-2)
Supplement: Supplementary file 8 — Supplementary Software [file 41467_2020_17062_MOESM8_ESM.zip › sleep_project_demo/gui_layout_toolbox/layoutdoc/User_guide2_3.html]

Colorbars and legends


|  |  |
| --- | --- |
| **User\_guide** |  |

  

## 2.3: Colorbars and legends

(The code for this example can be found here:
[ view
| edit
| run ]
)

When using layouts to position axes that can also have a
colorbar or legend it is very important to group the axes
with its colorbar and legend by putting them inside a `uicontainer`.

The following example illustrates this.

#### Open a window

Open a new figure window and remove the toolbar and menus.

```
window = figure( 'Name', 'Axes legend and colorbars', ...
            'MenuBar', 'none', ...
            'Toolbar', 'none', ...
            'NumberTitle', 'off' );
```

#### Create the layout

The layout involves two axes side by side. Each axes is placed into a uicontainer so that the legend and colorbar are "grouped" with the axes.

```
hbox = uix.HBoxFlex('Parent', window, 'Spacing', 3);
axes1 = axes( 'Parent', uicontainer('Parent', hbox) );
axes2 = axes( 'Parent', uicontainer('Parent', hbox) );
```

#### Add decorations

Give the first axes a colorbar and the second axes a legend.

```
surf( axes1, membrane( 1, 15 ) );
colorbar( axes1 );
  

theta = 0:360;
plot( axes2, theta, sind(theta), theta, cosd(theta) );
legend( axes2, 'sin', 'cos', 'Location', 'NorthWestOutside' );
```

  

|  |  |  |  |  |
| --- | --- | --- | --- | --- |
|  | Axes inside layouts | [Top] | Controlling visibility |  |

© 2018 The MathWorks Ltd
• Terms of Use
• Patents
• Trademarks
