# Supplementary material for: Astrocytic Ca2+ signaling is reduced during sleep and is involved in the regulation of slow wave sleep
Source: Nat Commun. 2020 Jul 6;11:3240. doi: 10.1038/s41467-020-17062-2 (PMC7338360; doi:10.1038/s41467-020-17062-2)
Supplement: Supplementary file 8 — Supplementary Software [file 41467_2020_17062_MOESM8_ESM.zip › sleep_project_demo/gui_layout_toolbox/layoutdoc/User_guide2_2.html]

Axes inside layouts


|  |  |
| --- | --- |
| **User\_guide** |  |

  

## 2.2: Axes inside layouts

(The code for this example can be found here:
[ view
| edit
| run ]
)

When using layouts to position axes, the position property
is set by the layout, not the user. Whether the `Position`
or `OuterPosition` property is used is determined by the
`ActivePositionProperty` property of the `axes`.
Note that the default setting is "outerposition".

The following example illustrates the two usages.

#### Open a window

Open a new figure window and remove the toolbar and menus.

```
window = figure( 'Name', 'Axes inside layouts', ...
            'MenuBar', 'none', ...
            'Toolbar', 'none', ...
            'NumberTitle', 'off' );
```

#### Create the layout

The layout involves two axes side by side. This is done using a flexible horizontal box. The left-hand axes is left with the `ActivePositionProperty` set to "outerposition", but the right-hand axes is switched to use `Position`.

```
hbox = uix.HBoxFlex('Parent', window, 'Spacing', 3);
axes1 = axes( 'Parent', hbox, ...
    'ActivePositionProperty', 'outerposition' );
axes2 = axes( 'Parent', hbox, ...
    'ActivePositionProperty', 'Position' );
set( hbox, 'Widths', [-2 -1] );
```

#### Fill the axes

Using `OuterPosition` (left-hand axes) is the normal mode and looks good for virtually any plot type. Using `Position` is only really useful for 2D plots with the axes turned off, such as images.

```
x = membrane( 1, 15 );
surf( axes1, x );
lighting( axes1, 'gouraud' );
shading( axes1, 'interp' );
l = light( 'Parent', axes1 );
camlight( l, 'head' );
axis( axes1, 'tight' );

imagesc( x, 'Parent', axes2 );
set( axes2, 'xticklabel', [], 'yticklabel', [] );
```

  

|  |  |  |  |  |
| --- | --- | --- | --- | --- |
|  | Position vs OuterPosition | [Top] | Colorbars and legends |  |

© 2018 The MathWorks Ltd
• Terms of Use
• Patents
• Trademarks
