# Supplementary material for: Astrocytic Ca2+ signaling is reduced during sleep and is involved in the regulation of slow wave sleep
Source: Nat Commun. 2020 Jul 6;11:3240. doi: 10.1038/s41467-020-17062-2 (PMC7338360; doi:10.1038/s41467-020-17062-2)
Supplement: Supplementary file 8 — Supplementary Software [file 41467_2020_17062_MOESM8_ESM.zip › sleep_project_demo/gui_layout_toolbox/layoutdoc/Function_reference2_6.html]

uix.VButtonBox


|  |  |
| --- | --- |
| **Function\_reference** |  |

  

## 2.6: uix.VButtonBox

  

Arrange buttons vertically in a single column

`obj` `= uix.VButtonBox(` `)`
:   is a type of VBox specialised for
    arranging a column of buttons, check-boxes or similar graphical
    elements. All buttons are given equal size and by default are
    centered in the drawing area. The justification can be changed as
    required.

`obj` `= uix.VButtonBox(` `prop``,` `value``,` `...` `)`
:   also sets one or more property values.

### uix.VButtonBox properties

| Property | Value | Description |
| --- | --- | --- |
| `BackgroundColor` | `colorspec` | Color to use for exposed areas of the layout background. This can be an RGB triple (e.g. [0 0 1]) or a colour name (e.g. 'b'). |
| `BeingDeleted` | on \| off | Deletion status. |
| `ButtonSize` | [w h] | The size for the buttons (all are given equal size). |
| `Contents` | empty GraphicsPlaceholder array \| array of graphics objects | Children within this layout, regardless of HandleVisibility. Note that this can only be set to permutations of itself. |
| `DeleteFcn` | `function_handle` | Function to call when the layout is being deleted. |
| `HorizontalAlignment` | left \| center \| right | The horizontal position of the buttons. |
| `Padding` | positive integer | Number of pixels of extra space around the outside of the layout. |
| `Parent` | empty GraphicsPlaceholder array \| figure \| container | Parent of the layout. |
| `Position` | [x y w h] | Position (x,y) and size (w,h) within figure or container. |
| `Spacing` | positive integer | Number of pixels of extra space to leave between elements in the layout. |
| `Tag` | string | Tag to associate with layout. |
| `Type` | string | Type of graphics object. |
| `Units` | inches \| centimeters \| normalized \| points \| pixels \| characters | Position units. |
| `VerticalAlignment` | top \| middle \| bottom | The vertical position of the buttons. |
| `Visible` | on \| off | Visibility. |

### For example:

```
f = figure();
b = uix.VButtonBox( 'Parent', f );
uicontrol( 'Parent', b, 'String', 'One' );
uicontrol( 'Parent', b, 'String', 'Two' );
uicontrol( 'Parent', b, 'String', 'Three' );
set( b, 'ButtonSize', [130 35], 'Spacing', 5 );
```

  
**See also:**

- `uix.HButtonBox` - for creating a horizontal arrangement of buttons
- `uix.VBox` - for creating a general vertical arrangement

  

|  |  |  |  |  |
| --- | --- | --- | --- | --- |
|  | uix.HButtonBox | [Top] | uix.Grid |  |

© 2018 The MathWorks Ltd
• Terms of Use
• Patents
• Trademarks
