# Supplementary material for: Astrocytic Ca2+ signaling is reduced during sleep and is involved in the regulation of slow wave sleep
Source: Nat Commun. 2020 Jul 6;11:3240. doi: 10.1038/s41467-020-17062-2 (PMC7338360; doi:10.1038/s41467-020-17062-2)
Supplement: Supplementary file 8 — Supplementary Software [file 41467_2020_17062_MOESM8_ESM.zip › sleep_project_demo/gui_layout_toolbox/layoutdoc/Function_reference1_3.html]

uix.BoxPanel


|  |  |
| --- | --- |
| **Function\_reference** |  |

  

## 1.3: uix.BoxPanel

  

Arrange a single element in a panel with boxed title

`obj` `= uix.BoxPanel(` `)`
:   creates a box-styled panel object with
    automatic management of the contained widget or layout. The
    properties available are largely the same as the builtin `UIPANEL`
    object. Where more than one child is added, the currently visible
    child is determined using the Selection property.

`obj` `= uix.BoxPanel(` `prop``,` `value``,` `...` `)`
:   also sets one or more property values.

### uix.BoxPanel properties

| Property | Value | Description |
| --- | --- | --- |
| `BackgroundColor` | `colorspec` | Color to use for exposed areas of the layout background. This can be an RGB triple (e.g. [0 0 1]) or a colour name (e.g. 'b'). |
| `BeingDeleted` | on \| off | Deletion status. |
| `BorderType` | none \| etchedin \| etchedout \| beveledin \| beveledout \| line | Type of border around the title and content areas. |
| `Contents` | empty GraphicsPlaceholder array \| array of graphics objects | Children within this layout, regardless of HandleVisibility. Note that this can only be set to permutations of itself. |
| `CloseRequestFcn` | `function_handle` | Function to call when the panel close icon is clicked. Note that if empty, no close button is shown. |
| `CloseTooltipString` | string | Custom tooltip string for close button. Note that if CloseRequestFcn is empty, no close button is shown. |
| `DeleteFcn` | `function_handle` | Function to call when the layout is being deleted. |
| `Docked` | logical | Is this panel in a docked state. See advanced maneuvers with panels for details. |
| `DockFcn` | function handle | Function to call when panel is docked or undocked. Note that if empty, no dock button is shown. See advanced maneuvers with panels for details. |
| `DockTooltipString` | string | Custom tooltip string for dock button (when panel is undocked). Note that if DockFcn is empty, no dock button is shown. |
| `FontAngle` | normal \| italic \| oblique | Title font angle. |
| `FontName` | string | Title font name (e.g. Arial, Helvetica etc). |
| `FontSize` | positive integer | Title font size. |
| `FontUnits` | inches \| centimeters \| normalized \| points \| pixels | Title font units for measuring size. |
| `FontWeight` | light \| normal \| demi \| bold | Title font weight. |
| `ForegroundColor` | `colorspec` | Title font color and/or color of 2-D border line. |
| `HelpFcn` | function handle | Function to call when the help icon is clicked. Note that if empty, no help button is shown. See advanced maneuvers with panels for details. |
| `HelpTooltipString` | string | Custom tooltip string for help button. Note that if HelpFcn is empty, no help button is shown. |
| `HighlightColor` | `colorspec` | 3-D frame highlight color. |
| `MaximizeTooltipString` | string | Custom tooltip string for minimize button (when panel is minimized). Note that if MinimizeFcn is empty, no minimize button is shown. |
| `Minimized` | logical | Is this panel in a minimized state. See advanced maneuvers with panels for details. |
| `MinimizeFcn` | function handle | Function to call when panel is minimized or maximized. Note that if empty, no minimize button is shown. See advanced maneuvers with panels for details. |
| `MinimizeTooltipString` | string | Custom tooltip string for minimize button (when panel is maximized). Note that if MinimizeFcn is empty, no minimize button is shown. |
| `Padding` | positive integer | Number of pixels of extra space around the outside of the layout. |
| `Parent` | empty GraphicsPlaceholder array \| figure \| container | Parent of the layout. |
| `Position` | [x y w h] | Position (x,y) and size (w,h) within figure or container. |
| `Selection` | positive integer or empty | Which child is visible. |
| `ShadowColor` | `colorspec` | 3-D frame shadow color. |
| `Tag` | string | Tag to associate with layout. |
| `Title` | string | Title string. |
| `TitleColor` | `colorspec` | color for the title bar background. |
| `Type` | string | Type of graphics object. |
| `UndockTooltipString` | string | Custom tooltip string for dock button (when panel is docked). Note that if DockFcn is empty, no dock button is shown. |
| `Units` | inches \| centimeters \| normalized \| points \| pixels \| characters | Position units. |
| `Visible` | on \| off | Visibility. See the visible example for more details. |

### For example:

```
f = figure();
p = uix.BoxPanel( 'Parent', f, 'Title', 'A BoxPanel', 'Padding', 5 );
uicontrol( 'Parent', p, 'Background', 'r' )
```

```
f = figure();
p = uix.BoxPanel( 'Parent', f, 'Title', 'A BoxPanel', 'Padding', 5 );
b = uix.HBox( 'Parent', p, 'Spacing', 5, 'Padding', 5  );
uicontrol( 'Style', 'listbox', 'Parent', b, 'String', {'Item 1','Item 2'} );
uicontrol( 'Parent', b, 'Background', 'b' );
set( b, 'Widths', [100 -1] );
p.FontSize = 12;
p.FontWeight = 'bold';
p.HelpFcn = @(x,y) disp('Help me!');
```

  
**See also:**

- `uix.Panel` - for creating a more standard panel
- `uix.CardPanel` - for creating an undecorated panel
- `uix.TabPanel` - for a panel with tabs for selecting the visible child

  

|  |  |  |  |  |
| --- | --- | --- | --- | --- |
|  | uix.CardPanel | [Top] | uix.TabPanel |  |

© 2018 The MathWorks Ltd
• Terms of Use
• Patents
• Trademarks
